# Demographical history and palaeodistribution modelling show range shift towards Amazon Basin for a Neotropical tree species in the LGM

**DOI:** 10.1186/s12862-016-0779-9

**Published:** 2016-10-13

**Authors:** Luciana Cristina Vitorino, Matheus S. Lima-Ribeiro, Levi Carina Terribile, Rosane G. Collevatti

**Affiliations:** 1 Laboratório de Genética & Biodiversidade, Instituto de Ciências Biológicas, (ICB), Universidade Federal de Goiás (UFG), Cx.P. 131, Goiânia, GO 74001-970 Brazil; 2 Laboratório de Macroecologia, Universidade Federal de Goiás (UFG), Campus Jataí, Cx.P. 03, Jataí, GO 75801-615 Brazil

**Keywords:** Bignoniaceae, Dry forest refugia, Ecological niche modelling, Phylogeography, Pleistocene arc hypothesis, Quaternary climatic changes

## Abstract

**Background:**

We studied the phylogeography and demographical history of *Tabebuia serratifolia* (Bignoniaceae) to understand the disjunct geographical distribution of South American seasonally dry tropical forests (SDTFs). We specifically tested if the multiple and isolated patches of SDTFs are current climatic relicts of a widespread and continuously distributed dry forest during the last glacial maximum (LGM), the so called South American dry forest refugia hypothesis, using ecological niche modelling (ENM) and statistical phylogeography. We sampled 235 individuals of *T. serratifolia* in 17 populations in Brazil and analysed the polymorphisms at three intergenic chloroplast regions and ITS nuclear ribosomal DNA.

**Results:**

Coalescent analyses showed a demographical expansion at the last *c*. 130 ka (thousand years before present). Simulations and ENM also showed that the current spatial pattern of genetic diversity is most likely due to a scenario of range expansion and range shift towards the Amazon Basin during the colder and arid climatic conditions associated with the LGM, matching the expected for the South American dry forest refugia hypothesis, although contrasting to the Pleistocene Arc hypothesis. Populations in more stable areas or with higher suitability through time showed higher genetic diversity. Postglacial range shift towards the Southeast and Atlantic coast may have led to spatial genome assortment due to leading edge colonization as the species tracks suitable environments, leading to lower genetic diversity in populations at higher distance from the distribution centroid at 21 ka.

**Conclusion:**

Haplotype sharing or common ancestry among populations from Caatinga in Northeast Brazil, Atlantic Forest in Southeast and Cerrado biome and ENM evince the past connection among these biomes.

**Electronic supplementary material:**

The online version of this article (doi:10.1186/s12862-016-0779-9) contains supplementary material, which is available to authorized users.

## Background

Recent phylogeographical works indicate expansion of South American seasonally dry forests (SDTF hereafter) across Quaternary glaciations [1, 2], agreeing with the idea of a unique and continuously distributed SDTF bordering Amazon Basin and northern Andes during glacial phases, a scenario also known as the Pleistocene Arc hypothesis (PLAH; [3]). The alternative scenario of range expansion towards the interior of Amazon Basin (the Pennington, Prado and Pendry hypothesis, PPPH hereafter; [4]) is also supported by phylogeographical patterns of other species, like the widely distributed *Tabebuia impetiginosa* [2]. Both hypotheses predict that the current disjunct distribution of SDTFs across South America is the result of vicariance (i.e. fragmentation) of a formerly more widespread and continuously distributed dry forest during the Last Glacial Maximum (LGM), which is known as the dry forest refugia hypothesis [5, 6].

However, the response of South American SDTF species to Quaternary climate change is still poorly understood, as revealed by contrasting phylogeographical patterns and multiple distribution dynamic response of species to Quaternary climate changes [1, 2, 7]. Actually, an increasing body of evidence does not support the generalized dry forest refugia hypothesis as originally proposed [7, 8]. For instance, the Bolivian Chiquitano dry forest was established only during the Holocene as a consequence of population expansions from southern Amazon rain forest [5, 6]. In Northeast Brazil, in Caatinga biome, the fossil records indicate that current climatic and vegetation conditions have been established only after 4.8 ka (thousands of years before present) [9], reaching as late as 1.0 ka in some regions [10]. Studies using ecological niche modelling (ENM) also show species-specific responses to the Quaternary climate changes [11]. Phylogenetic studies show an ancient origin of SDTFs in Mesoamerica, dated from 20 to 30 Ma (millions of years before present) [12], and to ~17 Ma in Brazilian Caatinga [13], whereas the fossil records suggest that SDTFs have originated around 13 to 12 Ma, at least in some part of its distribution [14, 15]. Dated phylogenies indicate a more ancient origin for Neotropical rain forests in the Cretaceous [16], although fossil records do not show evidence for Neotropical rain forests before the early Tertiary (~60 Ma) [15]. Such contrasting information reinforces the need for comprehensive studies about the dynamics of SDTF as an important source of evidence for the effect of climate change on species distribution.

SDTFs are one of the most threatened ecosystems in the world [17]. Although they originally occupied ~ 42 % of the tropical and subtropical forest regions [18], currently most of their remaining areas are in South America (~ 54.2 %), mainly in Northeast and Central Brazil and in Southeast Bolivia, Paraguay and Northern Argentina (Additional file 1: Figure S1), representing ~ 22 % of the forested area in South America [18]. In Brazil, the SDTFs are distributed from the Northeast, in Caatinga (Additional file 1: Figure S1), towards the Southwest in Misiones nucleus (that includes the northeastern Argentina and eastern Paraguay), and also are scattered throughout other vegetation types such as Amazon rain forest and savannas in Central Brazil [19] in areas of eutrophic and oligotrophic soils with neutral or quasi-neutral pH values and low levels of aluminium [20]. Most remaining areas of SDTFs in Brazil are threatened mainly by agricultural expansion, harvesting for wood products and the increase of fire frequency due to agricultural practices [11].

The distribution of SDTF species and the contrasting palaeoscenarios raise questions about the long-term stability and dynamics of the SDTF communities and the role of the Quaternary climate oscillations in the current patterns of genetic diversity and geographical distribution of plant species, that may be answered using multi-model inference approach [2, 21–23], as we address here for the widely distributed Neotropical tree *Tabebuia serratifolia* (Vahl) Nichols. (Bignoniaceae). *Tabebuia serratifolia* has a disjunct distribution and local low abundance throughout the SDTFs of South America (see Additional file 1: Figure S2). It occurs from the fragments of SDTFs in Northeast Brazil, in the Caatinga biome, towards the Misiones nucleus (Southwest Brazil, Bolivia), Paraguay and Peru, and in SDTFs of Amazon Basin and of Atlantic Forest. It is also scattered throughout the fragments of SDTFs in Central Brazil and the east slopes of Andes. *Tabebuia serratifolia* and *T. impetiginosa* are the most logged species in Brazil and the second most expensive timber, popularly known as ‘pau d’arco’ [24].

Here we studied the phylogeography of *T. serratifolia* and tested the dry forest refugia hypothesis concerning specific climatic oscillations during the last glacial cycle. Our analyses followed the framework proposed by Collevatti et al. [2, 21, 23], which is based on coalescence simulations of alternative demographical hypotheses based on biogeographical *a priori* hypotheses (PLAH and PPPH) and other two demographical expectations predicted by the ecological niche models (ENM). Hypotheses from the ENMs include the combined dynamics of both PLAH and PPPH hypotheses and range retraction at the LGM (instead of range expansion, a formerly widespread and continuously distributed SDTFs, as expected by the previous hypotheses). These hypotheses were tested elsewhere for *T. impetiginosa* [2].

## Results

### Genetic diversity and population structure

The sequencing of the chloroplast intergenic spacers *psbA-trnH, trnC-ycf6* and *trnS-trnG* generated combined data with 1,742 bp sites (excluding microsatellites and coding indels as one evolutionary step), 157 polymorphic sites and 79 different haplotypes for the 257 individuals of *T. serratifolia*. For ITS1 + 5.8S + ITS2 (ITS) we obtained a fragment of 518 bp with 23 polymorphic sites and 21 different haplotypes.

Chloroplast genome showed higher haplotype diversity (*h* = 0.873) than nuclear ITS (*h* = 0.772), but nucleotide diversity at cpDNA (π = 0.0032, SD = 0.0017) was lower than for nuclear genome (π = 0.0058, SD = 0.0034, Table 1). Higher genetic diversity was found for populations ALT, CRA, PNI and SAB.

**Table 1:** Genetic diversity based on Arlequin Ver 3.11 software and demographical parameters based on coalescent analysis performed with Lamarc 2.1.9 software for 17 populations of *Tabebuia serratifolia*

		cpDNA				ITS				Combined cpDNA and ITS nrDNA			
Pop	*N*	*k*	*h*	*π*	SD	*k*	*h*	*π*	SD	*θ*	*θ - 95 % CI*	*Ne*	*Ne - 95 % CI*
North - Northeast													
CCM	20	2	0.100	0.0001	0.0001	1	0.000	0.0000	0.0000	0.0001	0.0001 – 0.0004	270.34	270.34 – 1081.37
CRA	09	9	1.000	0.0065	0.0037	2	0.389	0.0075	0.0047	0.0071	0.0023 – 0.0231	19194.38	6217.90 – 62449.31
POF	27	8	0.752	0.0012	0.0008	3	0.145	0.0003	0.0005	0.0009	0.0005 – 0.0024	2433.09	1351.72 – 6488.24
SEC	04	2	0.500	0.0009	0.0008	3	0.833	0.0035	0.0030	–	–	–	–
Central – Central West													
ALT	32	16	0.845	0.0022	0.0012	2	0.405	0.0008	0.0008	0.0071	0.0001 – 0.0229	19194.38	270.34 – 61908.62
ARA	13	4	0.423	0.0008	0.0006	2	0.385	0.0007	0.0008	0.0006	0.0001 – 0.0019	1622.06	270.34 – 5136.52
BOD	10	4	0.533	0.0005	0.0004	1	0.000	0.0000	0.0000	0.0003	0.0001 – 0.0011	811.03	270.343 – 2973.78
GSV	03	1	0.000	0.0000	0.0000	2	1.000	0.0039	0.0036	–	–	–	–
LUZ	30	4	0.356	0.0008	0.0006	2	0.131	0.0003	0.0004	0.0006	0.0002 – 0.0013	1622.06	540.69 – 3514.46
MIM	13	7	0.846	0.0031	0.0018	5	0.756	0.0090	0.0004	0.0024	0.0009 – 0.0068	6488.24	2433.09 – 18383.35
POT	02	2	1.000	0.0040	0.0043	1	0.000	0.0000	0.0000	–	–	–	–
PNA	04	2	0.500	0.0003	0.0004	1	0.000	0.0000	0.0000	–	–	–	–
Southeast													
CAP	23	8	0.629	0.0010	0.0006	1	0.000	0.0000	0.0000	0.0008	0.0005 – 0.0014	2162.75	1351.72 – 3784.81
PNI	21	11	0.871	0.0032	0.0018	6	0.758	0.0080	0.0048	0.0038	0.0014 – 0.0124	10273.05	3784.81 – 33522.57
SAB	06	4	0.866	0.0047	0.0029	2	0.533	0.0010	0.0011	0.0012	0.0004 – 0.0117	3244.12	1081.37 – 31630.17
SCA	21	7	0.500	0.0009	0.0006	2	0.095	0.0007	0.0008	0.0009	0.0004 – 0.0016	2433.09	1081.37 – 4325.49
SUM	19	3	0.205	0.0009	0.0006	3	0.298	0.0022	0.0017	0.0005	0.0002 – 0.0955	1351.72	540.69 – 258177.90
Mean	–	5.53	0.584	–	–	2.29	0.337	–	–	–	–	–	–
SD	–	3.99	0.306	–	–	1.40	0.335	–	–	–	–	–	–
Overall	257	79	0.873	0.0032	0.0017	21	0.772	0.0058	0.0034	0.0275	0.0221–0.0355	74344.42	59745.88 – 95971.88

*N* sample size, *k* - number of haplotypes, *h* haplotype diversity, *π* nucleotide diversity, *SD* standard deviation, *θ* coalescent or mutation parameter, *Ne* effective population size; 95 % CI is the credibility interval at 95 %. Θ and *Ne* were estimated for combined cpDNA and ITS data

**Fig. 1:**
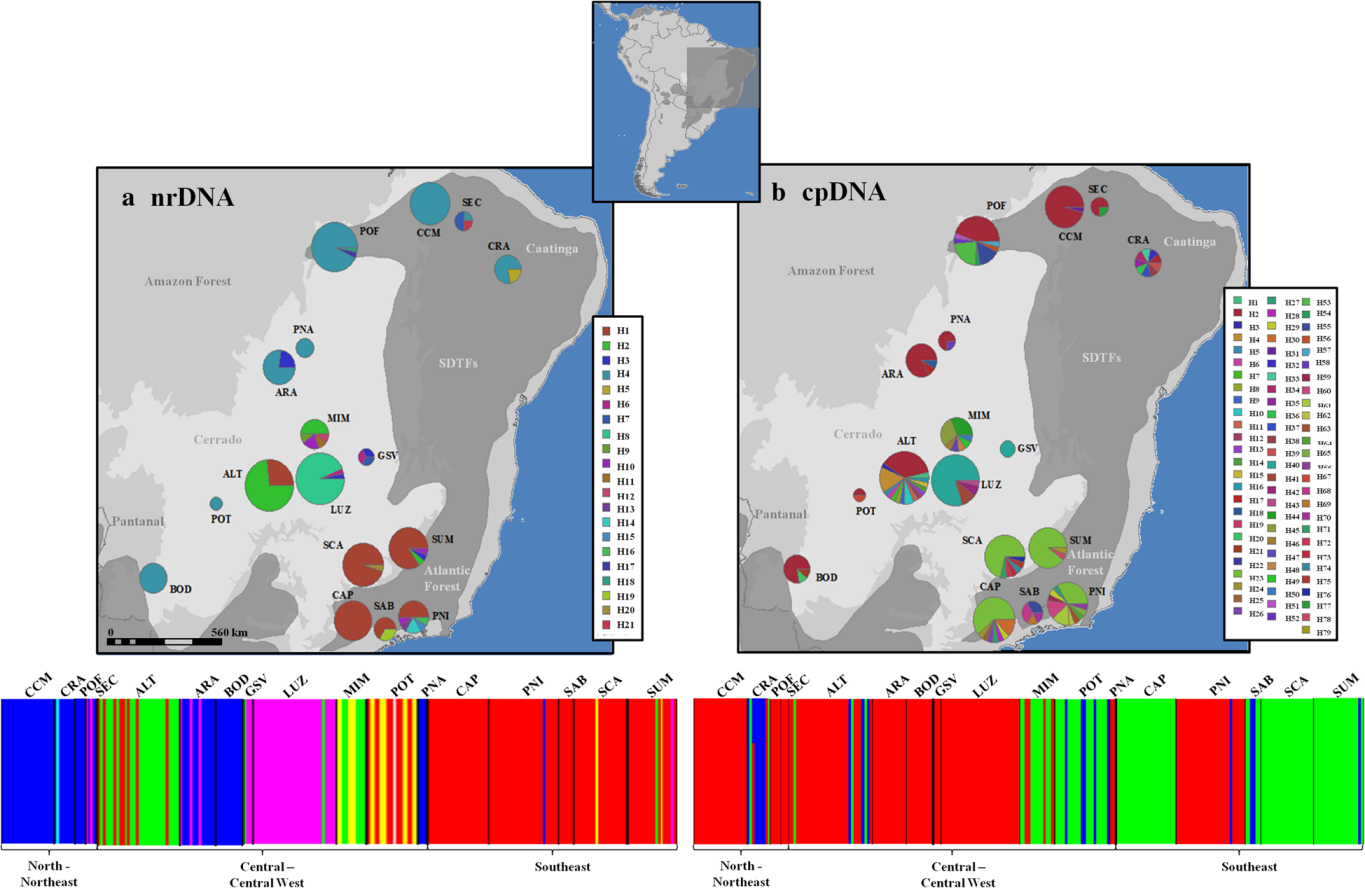
Geographical distribution of haplotypes of *Tabebuia serratifolia* and Bayesian clustering for (**a**) ITS and (**b**) cpDNA*,* based on the sequencing of 257 individuals from 17 populations. Different colours were assigned for each haplotype according to the figure legend. The circle size represents the sample size in each population and the circle sections represent the haplotype frequency in each sampled population. For details on population codes and localities see Additional file 2: Table S1. For BAPS clustering, each colour represents an inferred cluster (5 clusters for ITS and 3 for cpDNA)

Nuclear ITS showed four widespread haplotypes shared by populations from the Northeast and the Central Brazil (Fig. 1a, b) and cpDNA showed only two widespread haplotypes. Haplotype H4 (nrDNA ITS) and H2 (cpDNA) were shared by populations CRA, SEC, CCM, POF, from northeast, PNA and ARA, from central and POT and BOD, from Central-West Brazil (Fig. 1a and b). Despite haplotype sharing, the network showed evidence of genetic structure with high differentiation between populations from Northeast and Southeast Brazil (Additional file 1: Figure S3).

Analysis of Molecular Variance also showed a high differentiation among populations for both chloroplast (*ϕ*_*ST*_ = 0.528, *p* < 0.001) and ITS (*ϕ*_*ST*_ = 0.742, *p* < 0.001). Pairwise *ϕ*_*ST*_ was high between almost all population pairs for both cpDNA and ITS (Additional file 2: Table S6).

Bayesian clustering for cpDNA indicated an optimal partition of 3 groups for cpDNA and 5 groups for ITS (Fig. 1a, b). For cpDNA (Fig. 1b), populations from Northeast and Central-West were clustered together in one group (red cluster, ALT, ARA, BOD, CCM, GSV, LUZ, POF, PNA, SEC) with population PNI from Southeast. Populations from Southeast and Central-East formed other cluster (green, CAP, MIM, POT, SAB, SCA, SUM) with some individual from population ALT and CRA, from the Northeast. The third cluster (blue) was formed mainly by a population from the Northeast (CRA) and individuals from ALT, PNI, POT and PNA. ITS Bayesian clustering (Fig. 1a) split the cpDNA cluster 1 (red) in two. The first cluster comprised populations from North–Northeast and Central-West (blue, ARA, BOD, CCM, CRA, PNA, POF, SEC). The second cluster was formed by populations from Central-West Brazil (green, ALT, MIM). Populations from the Southeast were grouped in one cluster (red, CAP, PNI, SAB, SCA, SUM) with some individuals from populations of Central-West (ALT, POT). Population LUZ, from Central-West formed one cluster (pink) with individuals from populations ARA, SEC and SUM. Another cluster (black) was formed by individuals from different populations from the three geographical regions (Fig. 1a).

### Demographical history and time to most recent common ancestor

**Fig. 2:**
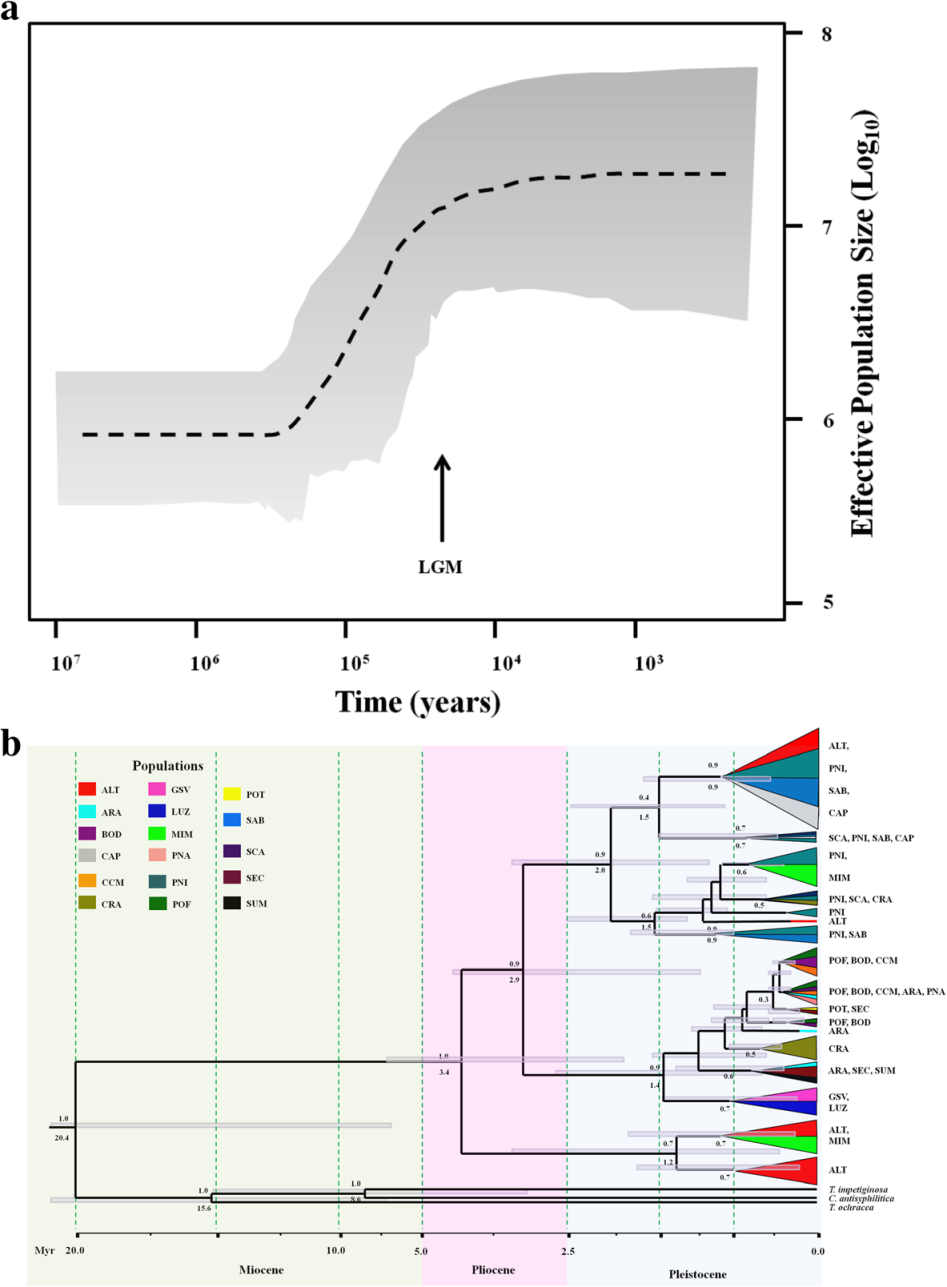
Demographical and evolutionary history of *Tabebuia serratifolia* lineages, based on concatenated sequences of cpDNA and ITS nrDNA. **a** Extended Bayesian Skyline Plot showing effective population size increase at *c*. 150 ka. **b** Coalescent tree showing that most lineage divergences occurred after the Lower Pleistocene. Tip section colour corresponds to population, following the figure legend. The section size corresponds to the number of haplotypes in each population in each clade. Grey bar corresponds to 95 % credibility interval of the mean time to the moat common ancestor; numbers above the branches are the support to the node (posterior probability); numbers below the branches are the node dating (time to the moat common ancestor). Time scale is in millions of years (Ma) before present

Extended Bayesian Skyline Plot (EBSP) analysis showed a demographical expansion of *T. serratifolia* at *c*. 130 ka (Fig. 2a) steadying after the LGM. We also found low values of mutation parameter *θ* for all populations (Table 1) and overall population (*θ* = 0.0275). Most populations had high effective population sizes (Table 1). Gene flow among all population pairs was negligible (less than 1 migrant per generation, Additional file 2: Table S7 and S8).

Haplotypes of *T. serratifolia* showed an ancient time to most recent common ancestor (TMRCA, Fig. 2b), ~3.4 Ma (95 % CI = 1.9 – 6.8 Ma), which coincides with the coalescence of haplotypes from populations ALT and MIM with all other haplotypes. Most divergences occurred after *c.* 1.5 Ma (Fig. 2b) and resulted in incomplete lineage sorting, with geographically distant populations sharing haplotypes and common ancestors.

### Setting-up demographical hypotheses

#### Species palaeodistribution modelling

**Fig. 3:**
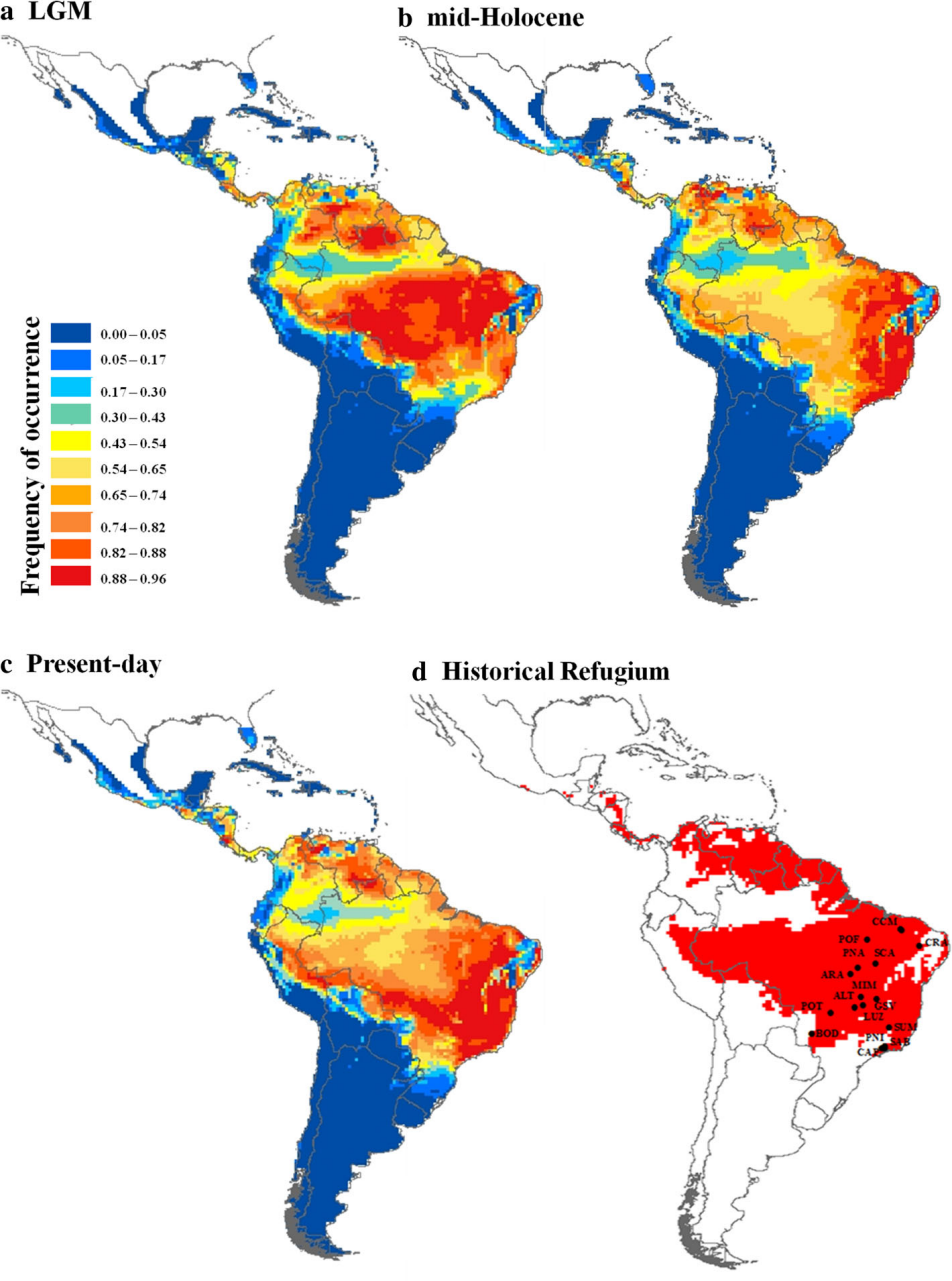
Maps of consensus of the 60 models expressing the ensemble potential distribution for *Tabebuia serratifolia*, based on ecological niche modelling. Potential distribution across the Neotropics during the (**a**) LGM (21 ka), (**b**) mid-Holocene (6 ka), (**c**) present-day (**d**) historical refugium through time (from the LGM to present-day)

The ENM showed that the range size of *T. serratifolia* at the LGM (Fig. 3a) was greater than at the mid-Holocene (Fig. 3b), but similar to the present-day (Fig. 3c, see also Additional file 1: Figure S4). During the LGM, the species was predicted to occur across the Amazon Basin, Brazilian Cerrado and western Caatinga. A range shift towards the Atlantic coast at the Northeast and the Southeast was predicted from the LGM to the mid-Holocene (Fig. 3, Additional file 1: Figure S4). In the present-day, *T. serratifolia* remained in the Atlantic Forest, but expanded its distribution towards the west Brazil, Bolivia and Peru, mainly bordering the Amazon Basin (Fig. 3, Additional file 1: Figure S4). The ensemble predicted a historical refugium extending from Amazon Basin towards (Fig. 3d) the Central and Southeast Brazil.

The analysis of uncertainty using hierarchical ANOVA showed lower proportional variance through time than among model methods (Additional file 2: Table S9). However, variation was highly spatially structured (Additional file 1: Figure S5) with higher proportion of variation from time component and low from ENM and AOGCMs (atmosphere-ocean general circulation models) within the geographical range of *T. serratifolia,* indicating that the ENMs were able to detect the effects of climate changes on the distribution dynamics of *T. serratifolia* through the last glaciation, despite the AOGCM variation (Additional file 1: Figure S5).

#### Inferring demographical hypotheses and model selection

Among the 60 palaeodistribution maps the pattern supported was PPPH (67 %), followed by “Both” hypothesis (PLAH + PPPH) with 15 % of maps, and “Range Retraction” (10 %). No ENM prediction supported the scenario expected by PLAH hypothesis (Additional file 2: Table S10) and 8 % of the maps matched none of the hypotheses.

Similarly, the coalescent simulations of alternative demographical hypotheses suggested that the hypothesis PPPH (Table 2) was the most likely scenario explaining the current pattern of genetic diversity at the chloroplast genome of *T. serratifolia* compared to the other competing hypotheses, using either two-tailed probability or Akaike criteria (*ΔAIC* and *AICw*; Table 2). For ITS, although hypothesis PPPH was the most likely for haplotype and nucleotide diversities (*ΔAIC* = 0.000 for both), hypotheses “Both” and “Range Retraction” could not be rejected for haplotype diversity (Table 2).

**Table 2:** Comparison of the demographical hypotheses in retrieve the haplotype (*h*) and nucleotide (*π*) diversities observed

	Hypothesis	*P*	*Δ AIC*	*AICw*	*P*	*Δ AIC*	*AICw*
			*h*			*π*	
cpDNA	PLAH	0.040	6.680	0.024	0.020	2.681	0.164
	PPPH	0.360	0.000	0.680	0.100	0.000	0.629
	Both	0.080	2.000	0.277	0.020	2.756	0.158
	Retraction	0.200	7.187	0.019	0.002	5.095	0.049
ITS	PLAH	0.194	2.852	0.111	0.040	2.000	0.241
	PPPH	0.498	0.000	0.465	0.140	0.000	0.587
	Both	0.194	1.287	0.243	0.020	3.428	0.106
	Retraction	0.378	1.876	0.181	0.040	4.382	0.066

Data obtained from 2,000 simulations using the software BayeSSC. (*P*) two-tailed probability. ***∆*** *AIC* and *AICw* Akaike Information Criterion delta and weights. *PLAH*, Pleistocene Arc Hypothesis, *PPPH*, Amazon SDF Hypothesis; Both, pattern matching both hypotheses PLAH + PPPH; Retraction, pattern matching a strong range retraction. See Fig. 5 for details about the demographical scenarios. *h* haplotype diversity, *π* nucleotide diversity

### Spatial patterns of genetic diversity

Genetic differentiation among populations and geographical distance were not significantly correlated for cpDNA (Mantel Test, r^2^ = 0.0223, *p* = 0.560) but significantly correlated for ITS (*r* ^2^ = 0.1892, *p* = 0.02).

**Fig. 4:**
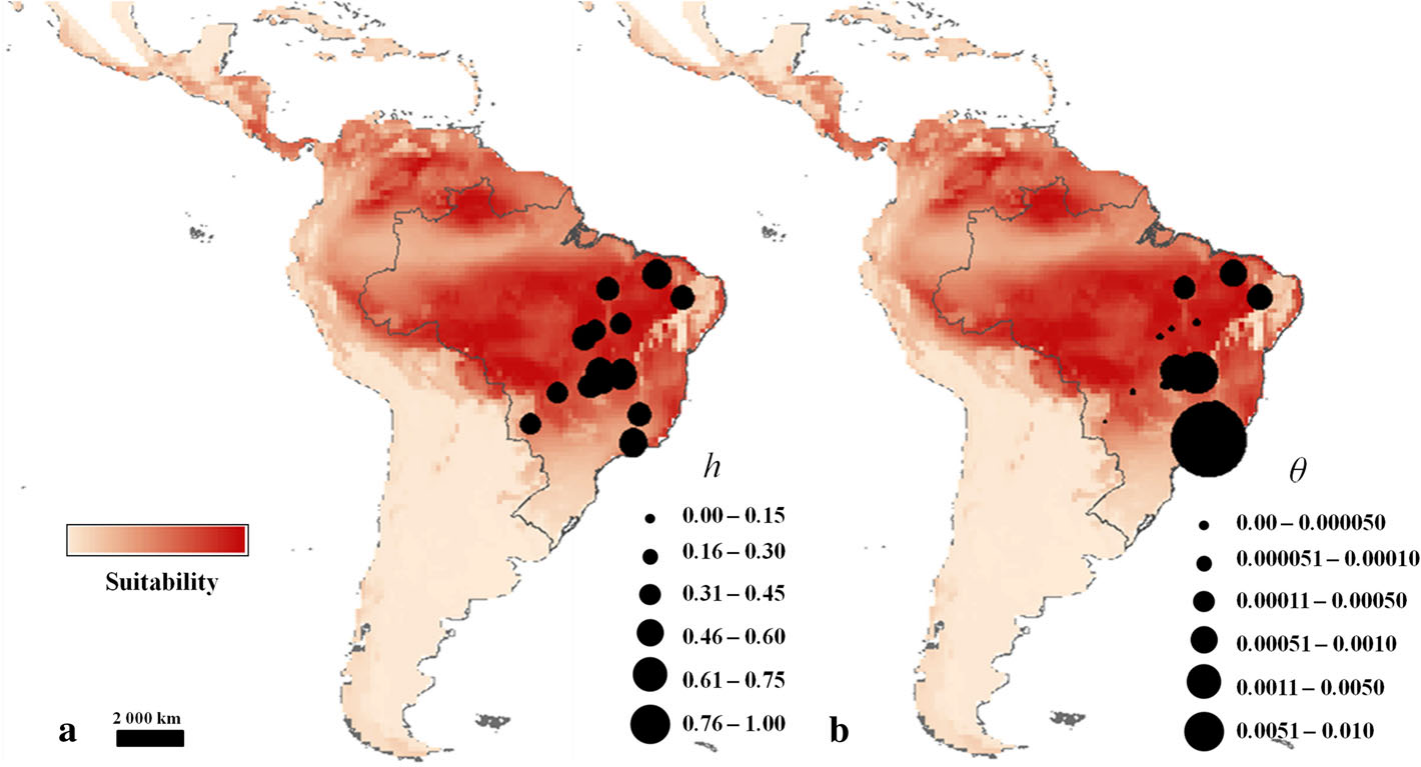
Spatial distribution of genetic diversity for ITS nuclear ribosomal DNA for *Tabebuia serratifolia,* in relation to the potential palaeodistribution at 21 ka. **a** Distribution of the haplotype diversity (*h*). **b** Distribution of the mutation parameter theta (*θ*). Circumference sizes are proportional to the value of genetic parameter, following the figure legends. The maps represent the consensus of the 60 models expressing the ensemble potential distribution for *Tabebuia serratifolia*, based on ecological niche modelling

Quantile regressions showed effects of climate changes on the genetic diversity for ITS. Populations in more climatically suitable areas and closer to the centroid of the distribution during the LGM showed higher haplotype (*h*) diversity (Fig. 4, see also Additional file 1: Figures S6 and S7). However, opposite relationships were observed for the mutation parameter *θ* (Additional file 1: Figures S5 and S6). In addition, populations in more unstable areas showed lower genetic diversity and lower *θ* (Additional file 1: Figure S8). Relationships for chloroplast genome were not significant.

## Discussion

Our findings from palaeodistribution modelling, phylogeographical analyses and coalescent simulations supported the hypothesis of dry forest refugia in South America for *T. serratifolia*. The pattern of genetic diversity is consistent with a demographical scenario of range expansion at the LGM, also supported by ENM, which predicted the scenario PPPH as the most likely; i.e. a range expansion and shift towards the Amazon Basin. Thus, the current disjunct distribution of *T. serratifolia* is most likely due to the range contraction of an ancient wider distribution, representing a climatic relict of drier and colder ice ages in Central-Southeast Brazil and Amazon Basin.

In fact, our results suggest that *T. serratifolia* was distributed northeastward during the LGM. Some typical species of SDTF are now widely distributed in Amazon Basin, occurring at low frequency in areas of more fertile soils [4], like *T. serratifolia* (see Additional file 1: Figure S2). The expansion of SDTFs throughout the Amazon Basin may have been favoured by the reduction in the sea level during the Pleistocene glaciations causing a decrease in the level of the Amazon Basin rivers, exposing areas for the colonization by SDTF species [4]. In addition, *T. serratifolia* is a more generalist species, currently occurring in forests of Amazon Basin, SDTFs and riparian forests of the Cerrado and Caatinga biomes and in Atlantic Forest. The suitable climatic conditions for *T. serratifolia* at present-day clearly show its preference for hot climates but with high variation in precipitation. Such climatic conditions were less available during the LGM at the Central Brazil (e.g. [25]) than during the mid-Holocene and the present-day. The temperature decrease during the LGM potentially lead to a range shift and expansion towards the Amazon Basin in response to the decreasing availability of suitable conditions in certain regions, such as the Southeast Brazil. In addition, although Amazon Basin remained forested during the LGM, the forest structure and species composition might have changed due to the lower temperatures, precipitation and atmospheric CO_2_ concentrations [26]. The southern Amazon forest became more seasonal, opening the opportunity for seasonally dry forest species colonization.

Despite the geographic and demographical expansion during glaciations, some populations had low haplotype and nucleotide diversity, contrary to the expected for a species with wide and continuous distribution during the LGM and with high effective population sizes. This is most likely due to the cycles of range shifts towards the Amazon Basin during recurrent glaciations that may have caused the extinction of haplotypes in some populations and a spatial assortment decreasing genetic diversity [27–29]. Indeed, quantile regressions showed a cline variation in the genetic diversity for ITS, because populations in more stable areas through time and with higher suitability had higher genetic diversity. Postglacial range shift towards the southeast and east may have led to spatial genome assortment due to leading edge colonization, as the species tracked suitable environments [29]. The leading edge colonization may have triggered the lower genetic diversity in populations at higher distance from the centroid of the geographical distribution at 21 ka, i.e. areas with lower climatic stability. The spreading from the leading edge may lead to bottlenecks of the colonizing genome, decreasing genetic diversity in some new colonizing areas [29]. In addition, allele surfing, i.e. the spread and frequency increase of a low-frequency allele that migrates on the wave of advance of a population in expansion [27, 28], and density-dependent processes due to the fast colonization and founder events may also cause patches and sectoring in genetic diversity [30, 31].

*Tabebuia serratifolia* showed slightly lower genetic diversity than *T. impetiginosa*, most likely due to different response to Quaternary climate changes. *Tabebuia impetiginosa*, showed a larger range expansion (PLAH + PPPH) during glacial periods (see [2]) as compared to *T. serratifolia* that showed a range shift towards the Amazon Basin (PPPH). Despite differences in effective population size and inheritance, chloroplast genome showed higher diversity than ITS nrDNA, which has four times the effective size of chloroplast genome and higher mutation rates. This may be due to concerted evolution in nrDNA that may homogenize copies decreasing genetic variation [32]. In addition, different genetic diversity signatures for ITS and chloroplast may be also due to different evolutionary rates corresponding to different time slices in the species evolution. The differences may also be due to the sequences sizes (1,743 versus 518 bp, for cpDNA and ITS respectively) because nucleotide diversity, which is corrected for sequence size, was slightly higher for ITS than for cpDNA.

Genetic diversity and number of haplotypes were higher in Central Brazil, in SDTFs from Cerrado biome (ALT, MIM), although some populations at Atlantic Forest also presented high genetic diversity (PNI). In fact, the results on ENM and quantile regressions showed that populations in more climatically suitable areas during the LGM presented higher genetic diversity (see Fig. 3) suggesting that stable areas at the Central Brazil were important refugia for *T. serratifolia.* Haplotype sharing between populations from Northeast Brazil (for instance, CRA in Caatinga) and populations in Atlantic Forest in Southeast Brazil and in Cerrado biome, and the Bayesian clustering of populations from these biomes (see Fig. 1) show the past connection between Caatinga and Cerrado and Atlantic Forest. Indeed, BAPS showed a more shallow genetic structure for cpDNA than for ITS, that may reflect differences in seed and pollen dispersal. In fact, genetic differentiation was higher for ITS (*ϕ*_*ST*_ = 0.742) than for cpDNA (*ϕ*_*ST*_ = 0.528), showing that restriction in pollen dispersal contributes more to differentiation than seed dispersal. BAPS showed almost the same result for both sequences for the Southeast cluster (green cluster for cpDNA and red cluster for ITS) except for population CRA in Northeast, that was not included in ITS red cluster. The pollen fossil record shows warmer and wetter climatic periods in the Late Pleistocene that may have caused the expansion of riparian forests in Cerrado biome connecting Atlantic and Amazon rainforests [33, 34]. Indeed, the ENM predictions for *T. serratifolia* show a high connection between the Atlantic Forest, Caatinga and Cerrado. However, the connection through the SDTF of the ‘dry diagonal’ may also have occurred [9, 35–37]. In Northeast Brazil, wet periods occurred during the last 210 ka, due to a southward displacement of the Intertropical Convergence Zone [37] and may have affected rainforest distribution [38]. The fossil record shows a forest expansion during the intermittent wet intervals that may have linked Amazon and Atlantic rainforests [39]. In addition, there are evidences of a drier climate in Atlantic Forest domain, in Southeast Brazil, c. 20 ka to 14 ka with establishment of species from Atlantic Forest only after that [40]. In other localities in Southeast Brazil, drier and colder climate may have persisted c. 48 – 18 ka [40, 41].

*Tabebuia serratifolia* lineages from Central Brazil (ALT, MIM) started to diverge first, in the Pliocene *c.* 3.4 Ma. However, major divergences occurred in the Lower Pleistocene (Calabrian Stage, 1.8 Ma to 781 ka [42]). We hypothesize that favourable climatic conditions for *T. serratifolia* (i.e. hot and wetter climates) were spatially more restricted before the Pleistocene leading to smaller effective population sizes as predicted by EBSP. Because the probability of coalescence is inversely related to the number of gene copies [43], most coalescence may occur just before demographical expansion, when populations have smaller effective population sizes (see [30] for a review). In fact, most coalescence occurred before the last glaciation coinciding with low population sizes showed by EBSP. Thus, the range expansion during the LGM, indicated by the palaeodistribution modelling and by the simulation of demographical hypotheses, matches the regional process of differentiation indicated by the coalescent tree with no later secondary contact. This result suggests an ancient process of incomplete lineage sorting due to expansion followed by a more regional population demographical expansion circumventing secondary contact and haplotype sharing, most likely due to restriction in soil suitability [2].

## Conclusions

In conclusion, our analyses based on coalescent simulation and ecological niche modelling strongly support a demographical and a geographical range expansion of *T. serratifolia* during the LGM as expected by dry forest refugia hypothesis. We also found a range shift towards the Amazon Basin, as expected by the prediction of PPPH scenario, with an important effect on the spatial pattern of genetic diversity. Finally, we showed here that phylogeographical analyses coupled with ecological niche modelling and coalescent simulations can be a very powerful framework for evaluating alternative hypotheses and potentially useful for disentangling mechanisms involved in the origin of the disjunct distribution of SDTFs.

## Methods

### Population sampling

We sampled 17 populations (257 individuals) of *T. serratifolia* mainly in SDTFs from Caatinga, Cerrado, Amazon and Atlantic Forest biomes (Fig. 1, see also the Additional file 2: Table S1). Distance between population pairs ranged from 13 (CCM and SEC) to ~2.276 km (CRA and BOD). In all populations we sampled expanded leaves or cambium from adult individuals for DNA extraction. Because of the high level of anthropic disturbs in the Brazilian SDTFs, some regions had limited amount of living individuals, resulting in different sample sizes among populations (Table 1). The DNA from leaves and cambium was extracted following the standard CTAB procedure [44].

We used samples of *Tabebuia ochracea, T. impetiginosa* and *Cybistax antisyphilitica* as outgroups for divergence dating (Additional file 2: Table S1). Sampling was not performed in conservation units and thus did not require any license. Vouchers were compared to herbarium material from the Federal Unversity of Goiás (Universidade Federal de Goiás), in Goiânia, Brazil.

### DNA sequencing

Polymorphism was assessed using sequences of three chloroplast DNA (cpDNA) intergenic spacers, *psbA-trnH, trnC*-*ycf6* and *trnS*-*trnG* [45], and the nrDNA ITS1 + 5.8S + ITS2 (primers 75 and 92, [46]). DNA was amplified by PCR following the same conditions described in Collevatti *et al*. [2] for *T. impetiginosa*. PCR products were sequenced on a GS 3500 Genetic Analyzer (Applied Biosystems, CA) using the BigDye® Terminator v3.1 Cycle Sequencing Kit (Applied Biosystems), according to the manufacturer instructions. All fragments were sequenced in forward and reverse directions.

Sequences were analysed and edited using the software SeqScape v3.0 (Applied Biosystems, CA) and final alignments were obtained using the software ClustalΩ [47]. Polymorphisms at mononucleotide microsatellites in cpDNA were excluded due to ambiguous alignment and to higher mutation rates. Long indels were coded as one evolutionary event (one character) and each base pair were equally weighted before analyses. The sequences of the three chloroplast regions were concatenated for all analyses.

### Genetic diversity and population structure

To understand the relationships among haplotypes of *T. serratifolia* we inferred intraspecific phylogeny for chloroplast and ITS data using median-joining network analysis implemented in the software Network 4.6.1.0 [48]. Genetic diversity for each population and overall populations were estimated based on nucleotide (π) and haplotype (*h*) diversities [49] using the software ArlequinVer 3.11 [50].

The hypothesis of population differentiation was tested based on an analysis of molecular variance (AMOVA, [51]) using the software ArlequinVer 3.11 [50], that estimates *ϕ*_*ST*_ *,* analogous to *F*_*ST*_, using information on the allelic content of haplotypes, as well as their frequencies [51]. Population structure was also assessed using Bayesian clustering implemented in the software BAPS v6.0 [52]. cpDNA and ITS were analysed as separate partitions with linkage model for sequences. We performed population admixture analysis based on mixture clustering with estimated number of clusters (K) with an upper limit of K = 17.

### Demographical history and time to most recent common ancestor

To trace the dynamics in effective population size we performed an Extended Bayesian Skyline Plot (EBSP) analysis [53] implemented in BEAST 1.8.3 [54], which calculates the effective population size (*Ne*) through time combining data from different partitions. Chloroplast and nrDNA ITS data were combined in one analysis, but separate priors were given for each partition. No evidences of heterozygous individuals were found when sequences were analysed using SeqScape v2.6 (Applied Biosystems, CA). Thus, recombination was neglected in all coalescent analyses. To set the priors, evolutionary model selection for both chloroplast and ITS regions was performed using Akaike information criterion (AIC), implemented in the software jModelTest2 [55]. For chloroplast regions, the model HKY + G was selected (-lnL = 3576.1766), with kappa = 1.5 and gamma shape equal to 0.0170. For ITS, the evolutionary model TIM1 + G was selected (-lnL = 905.4461) with gamma shape equal to 0.010. We used the relaxed molecular clock model (uncorrelated lognormal) for both chloroplast and ITS. Mutation rates for both chloroplast and ITS regions were the same used for the taxonomic related species, *T. impetiginosa* [2] and *T. aurea* [56]. Four independent analyses were run for 30 million generations. Convergence and stationarity were checked, and the independent runs were combined using the software Tracer v1.6 [57]. We considered the results only when ESS ≥ 200 (effective sample size).

Further, we estimated the mutation or coalescent parameter *θ* = 2*μN*_*e*_ (mutation parameter, *θ* = 4*μN*_*e*_ for diploid genome) based on a Bayesian modelling using Markov Chain Monte Carlo (MCMC) approach [58] implemented in Lamarc 2.1.9 software [59]. For this analysis, we excluded populations with less than 5 individuals (see Table 1). The analyses were run with 20 initial chains of 10,000 steps and three final chains of 100,000 steps. The chains were sampled every 100 steps. We used the default settings for the initial estimate of theta. The program was run three times to certify for convergence and validate the analyses using Tracer v1.6 [57] and combined results were then generated. We considered results when ESS ≥ 200 and when marginal posterior probability densities were unimodal and converged among runs. The effective population size was estimated from the mutation parameter *θ* using a generation time of 15 years (based on flowering time on permanent plots; RG Collevatti, unpublished data) and the same mutation rate used to related species [2, 56]. To study the past connectivity among populations we also estimated immigration parameter, M = 2*N*_*e*_ *m*/*θ* (immigration rate, M = 4*N*_*e*_ *m*/*θ* for diploid genome), using Lamarc 2.1.9 software.

TMRCA was estimated based on Bayesian coalescent analysis implemented in the software BEAST 1.8.3 [54]. For both chloroplast and ITS, a relaxed molecular clock (uncorrelated lognormal) was assumed. The ucld.stdev parameter (standard deviation of the uncorrelated lognormal relaxed clock) and the coefficient of variation were inspected for among branch rate heterogeneity within the data. In all runs the ucld.stdev was greater than 1.25 and the coefficient of variation frequency histogram viewed in Tracer abutted against zero (~1.6 to 2.5) showing heterogeneity among branches. We assumed population expansion, based on the Extended Bayesian Skyline Plot (EBSP) analysis [60]. Prior *N*_*e*_ was set to assume a lower bound from zero to infinity upper bound with exponential distribution. Four independent analyses were run for 100 million generations. Mutation rates for both chloroplast and ITS regions were the same used for a taxonomic related species [2, 56]. We also ran an empty alignment (sampling only from priors) to verify the sensitivity of results to the given priors. The analysis showed that our data are informative because posterior values (e.g. posterior probability) were different from those obtained from empty alignment (priors only).

### Setting-up demographical hypotheses

#### Species palaeodistribution modelling

Occurrence records of *T. serratifolia* across Neotropics (Additional file 1: Figure S2, Additional file 2: Table S1) were obtained from GBIF (Global Biodiversity Information Facility http://www.gbif.org/). All records were examined for probable errors and duplicates, and the nomenclature was checked for synonymies. The records were mapped in a grid of cells of 0.5° × 0.5° (longitude x latitude) encompassing the Neotropical region to generate a matrix of 698 presences (cells with occurrence records, Additional file 2: Table S2) used for distribution modelling (see below).

We also generate environmental layers as predictors for ENM using five bioclimatic variables (annual mean temperature, mean diurnal range, isothermality - mean diurnal range/temperature annual range, precipitation of wettest month, and precipitation of driest month) and subsoil pH (30-100 cm). These five bioclimatic variables present low multicollinearity and were selected by factorial analysis with Varimax rotation using the 19 bioclimatic variables obtained in the EcoClimate database (www.ecoclimate.org; [61]). The climate predictors present 0.5° of spatial resolution and were obtained for LGM (21 ka), mid-Holocene (6 ka) and pre-industrial (expressing the current climate) periods, using simulations from four atmosphere-ocean general circulation models (AOGCM): CCSM4, CNRM-CM5, MIROC-ESM, MPI-ESM-P and MRI-CGCM3 (Additional file 2: Table S3). Subsoil pH (30–100 cm) was obtained from Harmonized World Soil Database (version 1.1, FAO/IIASA/ISRIC/ISS-CAS/JRC 2009). We assumed subsoil pH to be constant through time (from LGM to pre-industrial) and used in ENM as a “constraint variable” to better model the environmental preferences of *T. serratifolia*.

The distribution of *T. serratifolia* was modelled using 12 methods encompassing both presence-only, presence-background and presence-absence algorithms (Additional file 2: Table S4). Because absence data are not available for *T. serratifolia*, we randomly selected pseudo-absences throughout the Neotropical grid cells (excepting cells with presences) keeping prevalence equal to 0.5 to calibrate the ENM based on presence-absence observations [62, 63]. This approach was based on studies suggesting that the extent of the geographical region in which the pseudo-absence points are taken have important influences for prediction and performance of ENM [64, 65]. Thus, selecting pseudo-absences throughout the distribution of *T. serratifolia* (i.e. the Neotropical region) essentially represents a compromise between generating models that do not generalize well, not produce over predictions of distribution areas ignoring important spatial structure associated with finer scale environmental gradients [65].

The distribution of *T. serratifolia* was first modelled for current (i.e. pre-industrial) climate and then projected onto LGM (21 ka) and mid-Holocene (6 ka) palaeoclimatic conditions. All ENMs were ran in the integrated computational platform BIOENSEMBLES [66] following the ensemble approach [67]. The procedures for modelling using the ensemble approach were extensively discussed elsewhere [2, 66] and just a brief description will be presented below. For each species distribution model, the occurrence points were randomly partitioned into two subsets (training and testing) comprising 75 and 25 % of dataset, respectively, and this procedure was repeated 50 times. Initial models were evaluated by True Skill Statistics (TSS, [68]); models with poor performance (TSS < 0.5) were eliminated (TSS values for all models are provided in Additional file 2: Table S5). Remaining models were combined (a weighted average by TSS value of each model) to generate the frequency of models supporting the occurrence of the species in each cell of Neotropical grid (i.e. consensus maps), for both current and past climatic layers. Next, the predictive maps for LGM, mid-Holocene and present-day were obtained by using the 10^th^ percentile lowest presence threshold, i.e. excluding 10 % of the lowest consensus values linked to a occurrence record used to build the ENM.

We applied a hierarchical ANOVA using the predicted suitability from all models (12 ENM × 5 AOGCMs × 3 Times) as a response variable to disentangle the effects of climate change on species distribution through the time from predictive uncertainties in the potential distribution due to modelling components (i.e. ENM, AOGCMs). For this, the ENM and AOGCM components were nested into the time component, but crossed by a two-way factorial design within each time period (see [69] for details about hierarchical design).

#### Inferring demographical hypotheses

The 60 predicted palaeodistribution maps (estimated frequencies of occurrence for the 12 ENMs × 5 AOGCMs) were visually inspected by two of us (RGC and LCV), using a double-blind experimental design, and classified as supporting the alternative scenarios following Collevatti et al. [2]: i) the ‘Pleistocene Arc Hypothesis’, PLAH hypothesis [3], an expansion throughout the Central and Southwest Brazil; ii) the ‘Amazon SDTF Hypothesis’, PPPH hypothesis [4], a westward range shift, toward the Amazon Basin; iii) PLAH + PPPH (“Both”), i.e. a prediction for the past distribution as expected by both hypotheses, resulting in an expansion throughout the Central and Southwest Brazil and also towards the interior of Amazon Basin; iv) “Range Retraction”, a retraction in geographical range in Central Brazil but without range shift. Although PLAH, PPPH and “Both” scenarios show different distribution dynamics for the SDTF in South America, they are all compatible with the dry forest refugia hypothesis.

#### Demographical history simulation

**Fig. 5:**
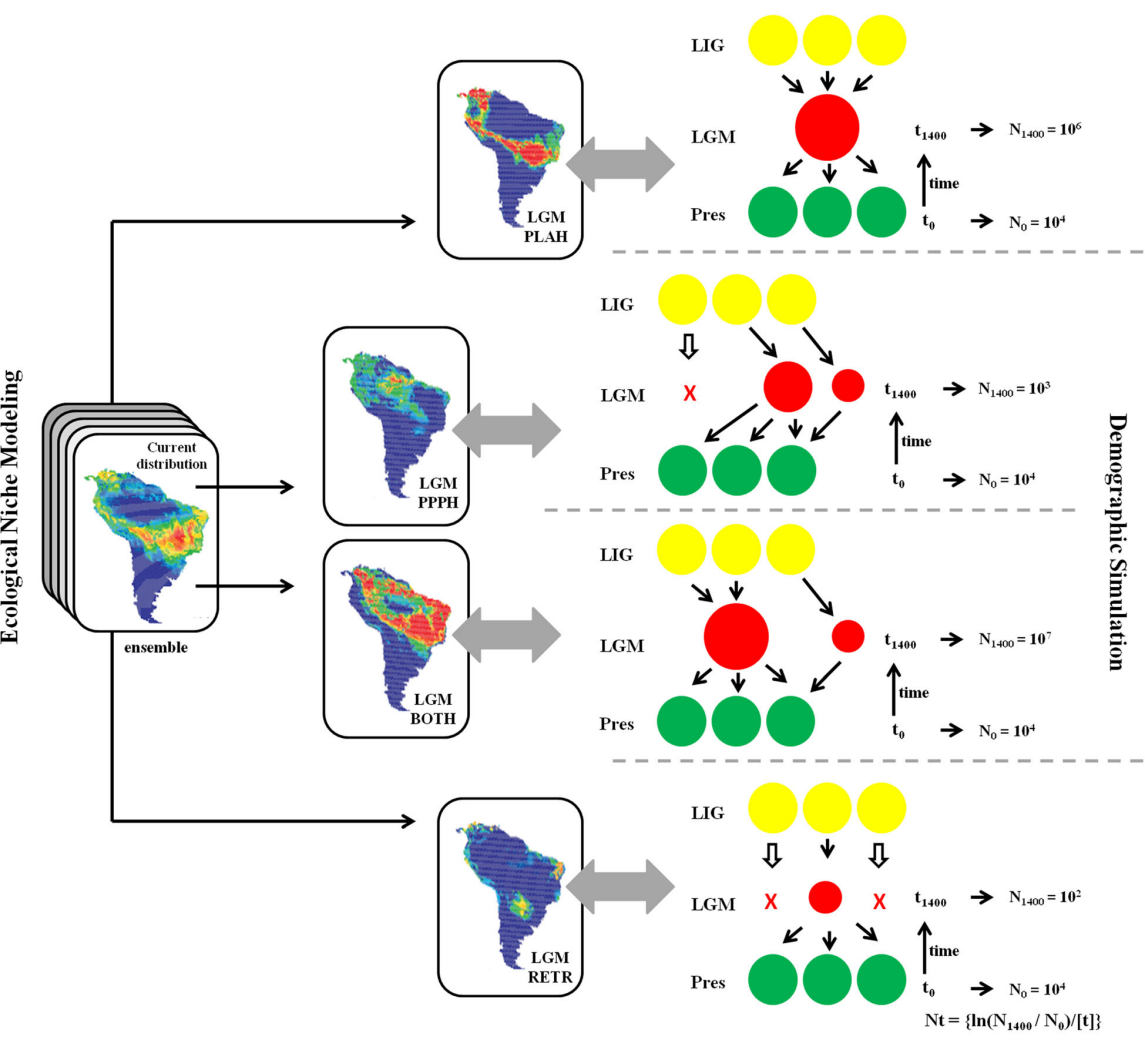
The demographical history scenarios simulated for *Tabebuia serratifolia* and their geographical representation. Circles represent the demes. The size and location of circle during the LGM indicate demographical population expansion or shrink, and geographical range shift at that time. LIG: last interglacial; LGM: last glacial maximum; Pres: present-day; N0: effective population size at time t0 (present); N1: effective population size at time t1400 (1,400 generations ago). The demographical scenarios correspond to: PLAH, Pleistocene Arc hypothesis; PPPH, the ‘Amazon SDF’ hypothesis; Both (PLAH + PPPH), i.e., an expansion throughout the Central and Southwest Brazil and also westward towards the Amazon Basin; Retraction, a retraction in geographical range in Central Brazil

The demographical history of *T. serratifolia* was modelled and simulated based on coalescent analysis [45] implemented in the software ByeSSC [70, 71]. We modelled four demographical scenarios (Fig. 5) according to the hypotheses supported by ecological niche modelling and biogeographical hypotheses (PLAH, PPPH, “Both”, or “Range Retraction”), following the framework described in Collevatti et al. [2, 11]. For each demographical scenario, we run 2,000 independent simulations for each sequence region. Model calibration was based on parameters estimated with Lamarc 2.1.9 software and the molecular evolution of the chloroplast non-coding and ITS regions, i.e. the same evolutionary model, sequence size (in base pair) and mutation rates. The number of generations until the LGM (at 21 kyr BP) was calculated using a generation time of 15 years (RG Collevatti unpublished data).

Demographical hypotheses were simulated backward, with 17 demes from t0 (present) to t1400 generations ago (at the LGM). Population sizes at time t were calculated from Nt = {ln(N1/N0)/[t]}, where N0 = 10,000 was the same for all scenarios, and N1 shifted among them according to our theoretical expectation (see Fig. 5 for details). In BayeSSC negative growth implies population expansion, because coalescent simulations run backward through time. Thus, a negative growth rate implies a population larger now than in the past, and a positive growth, a population smaller now than in the past. Because of the high variation in effective population sizes for *T. serratifolia* (see Table 1), we performed simulations with different initial deme sizes for N0 = 1,000, N0 = 10,000 and N0 = 100,000 for all scenarios. Simulations using N0 = 1,000 presented all values of haplotype and genetic diversity lower than those observed for *T. serratifolia* for all demographical hypotheses and N0 = 100,000 retrieved almost all values higher than the observed for *T. serratifolia*. Henceforth, we used simulations for N0 = 10,000.

To simulate migration we considered a finite island model in which all current demes are descendants from lineages originally in deme 1 at t generations ago, meaning that as the tree builds back through time, there is a 0.01/generation chance that each lineage in deme x will migrate to deme 1. We also simulated different values of migration rate but values < 0.01 were not sufficient to show any demographical variation at the time scale we are working and values > 0.1 retrieved equal likelihoods for all models. For PPPH and ‘Range Retraction’ we considered that each lineage in deme x will migrate to deme 1 and than shrink until extinction.

#### Model selection

Simulated alternative models were compared based on the distribution of haplotype and nucleotide diversities in the 2,000 simulations for both chloroplast and ITS sequences. We estimated two-tailed probabilities as twice the number of diversity estimates that were higher than the observed, divided by the number of simulations, so that a high P-value indicates failure to reject the model. We also estimated Akaike information criterion (*AIC*) for model choice. The log-likelihood, ln(L), was estimated as the product of the height of the empirical frequency distribution at the observed value of diversity by the maximum height of the distribution. *AIC* (-2Ln(L) + 2 K, where K is the number of free parameters, 2 for all models) was transformed into *AIC* weight of evidence (*AICw*), given by exp[-0.5(AIC – AICmin)] [72], from which we obtained *ΔAIC*; i.e. the difference of *AICw* between each model and the best model. Models with *ΔAIC* < 2 were considered as equally plausible to explain the observed pattern [73]. *AICw* was expressed as a relative value among models (see [72]).

### Spatial patterns in genetic diversity

Because ENM and simulation supported range shift towards Amazonia Basin (see Results) for *T. serratifolia,* we used spatially explicit analyses to detect spatial patterns in observed genetic diversity in response to late Quaternary climate oscillations, for both cpDNA and ITS. Spatial expansion may lead to gradients in genetic diversity because of allele surfing during the colonization of new areas and “lead trail” expansion (see [30, 31] for reviews).

We first tested if differentiation is an effect of isolation by distance [72]. Pairwise linearized *ϕ*_*ST*_ (analogous to *F*_*ST*_) among pairs of populations were estimated for both cpDNA and ITS and correlated with a geographical distance matrix (logarithm of geodesic distance) by a Mantel test using ArlequinVer 3.11 [52] and statistical significance was tested by a non-parametric permutation test (10,000 permutations).

We used quantile regressions to analyse the relationships of climatic suitability and stability through time with genetic diversity [73]. For this, we calculated the difference of ensemble suitability between LGM, mid-Holocene and present-day as a measure of climate stability through time. Next, we analysed whether historical changes in species’ geographical range generated a cline spatial pattern in genetic parameters, haplotype (*h*) and nucleotide (π) diversities, *Ne* and *θ* due to expansion of climatically suitable conditions. For this, we obtained the distance between each sampled population and the centroid of historical refugium and of the predicted distribution at 21 ka, 6 ka and present-day, and then we performed quantile regressions of genetic parameters against these spatial distances and against climatic stability.

## Additional files

Additional file 1: Figures S1–S8. with details on ecological niche modelling, networks and quantile regressions. (DOCX 3553 kb)
Additional file 2: Tables S1–S11. with sampling locations and details on ecological niche modelling and demographical parameters. (DOCX 143 kb)
