# Supplementary material for: Demographical history and palaeodistribution modelling show range shift towards Amazon Basin for a Neotropical tree species in the LGM
Source: BMC Evol Biol. 2016 Oct 13;16:213. doi: 10.1186/s12862-016-0779-9 (PMC5062830; doi:10.1186/s12862-016-0779-9)
Supplement: Additional file 1: Figures S1–S8. — with details on ecological niche modelling, networks and quantile regressions. (DOCX 3553 kb) [file 12862_2016_779_MOESM1_ESM.docx]

**Demographical history and palaeodistribution modelling show range shift towards Amazon Basin for a seasonally dry forest tree species in the LGM**

Luciana Cristina Vitorino, Matheus S. Lima-Ribeiro, Levi Carina Terribile, Rosane G. Collevatti

**Supporting Information - Figures**


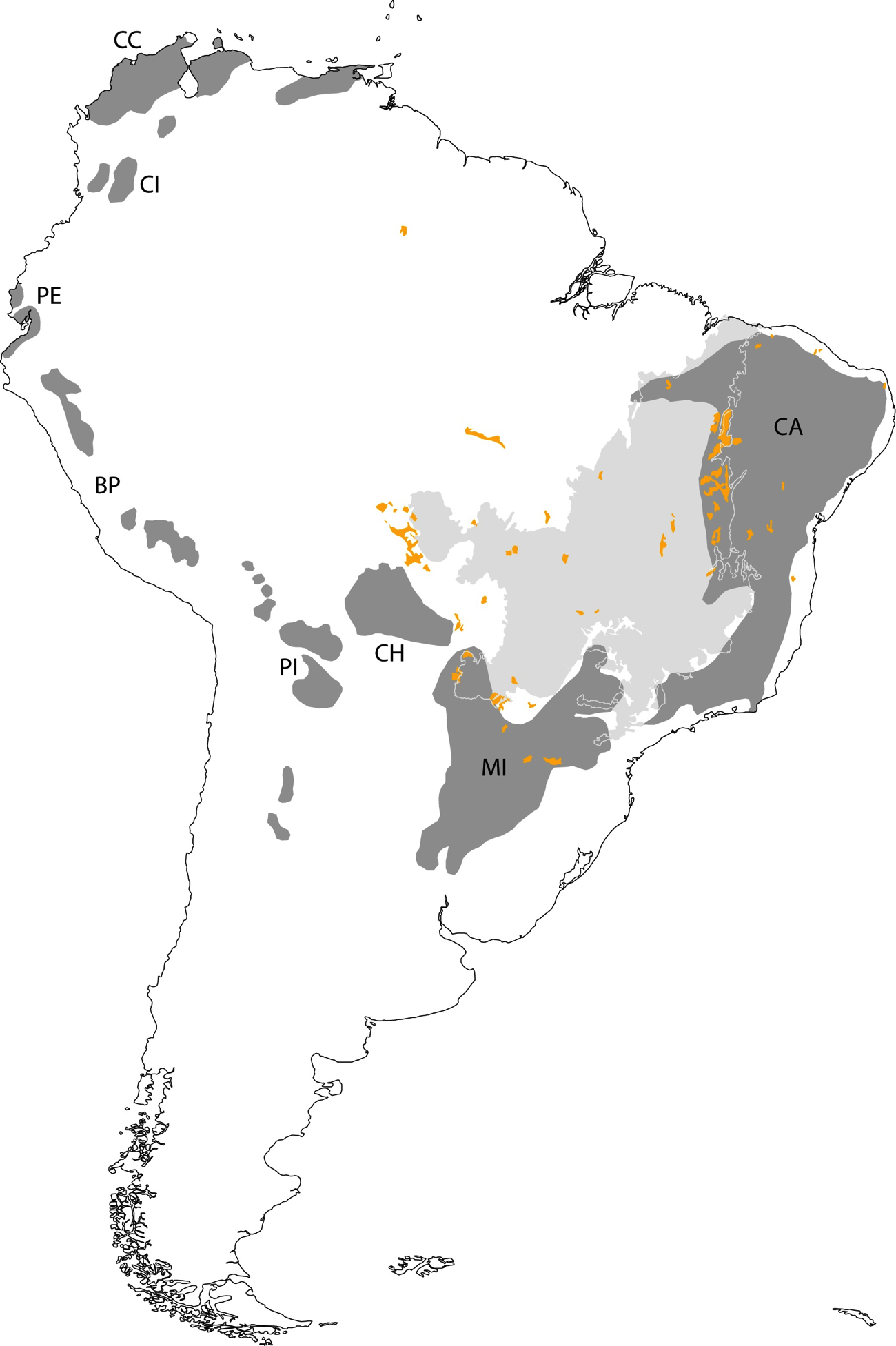


**Fig S1** Distribution of seasonally dry forest (SDTF) (dark grey areas) in South America (Collevatti et al. 2013). Orange areas represent disjunct areas of deciduous and semideciduous forest in Brazil (mapped from IBGE, 2011). CA, Caatinga nucleus; MI, Misiones nucleus; CH, Chiquitano Dry Forest nucleus; PI, Piedmont nucleus; BP, Bolivian and Peruvian inter-Andean valleys; PE, Pacific coastal Ecuador; CI, Colombian inter-Andean valleys; CC, Caribbean coast of Colombia and Venezuela. Light grey area represents the Brazilian Cerrado.


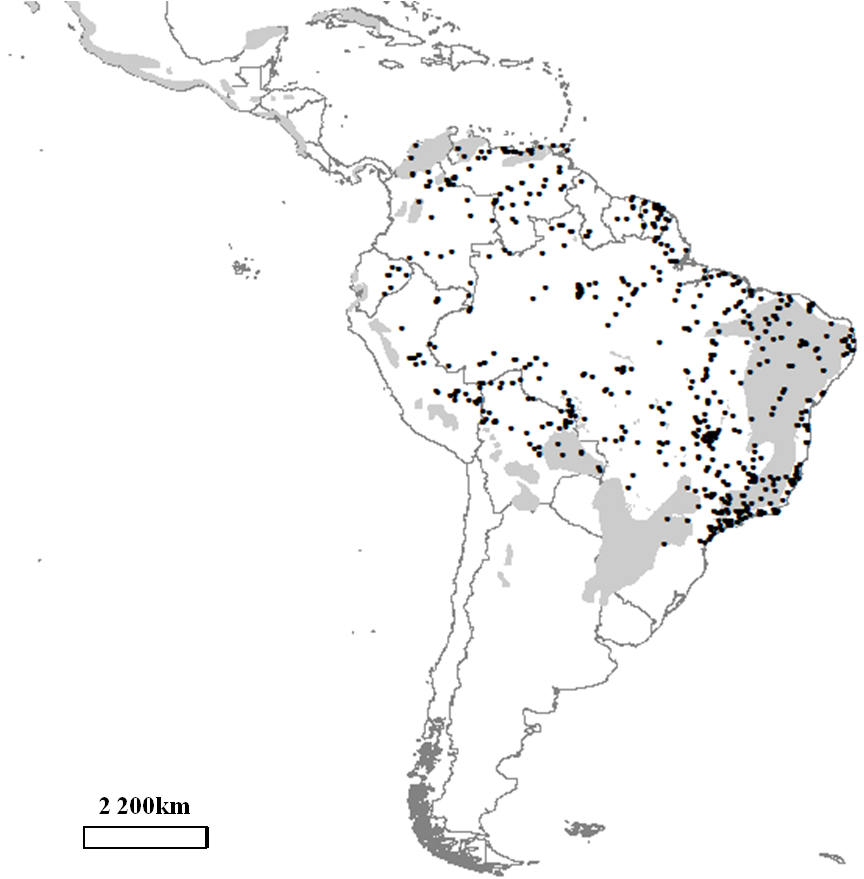


**Fig S2** Current geographical distribution of *Tabebuia serratifolia* across the Neotropics based on 698 occurrence records used in ecological niche modelling.


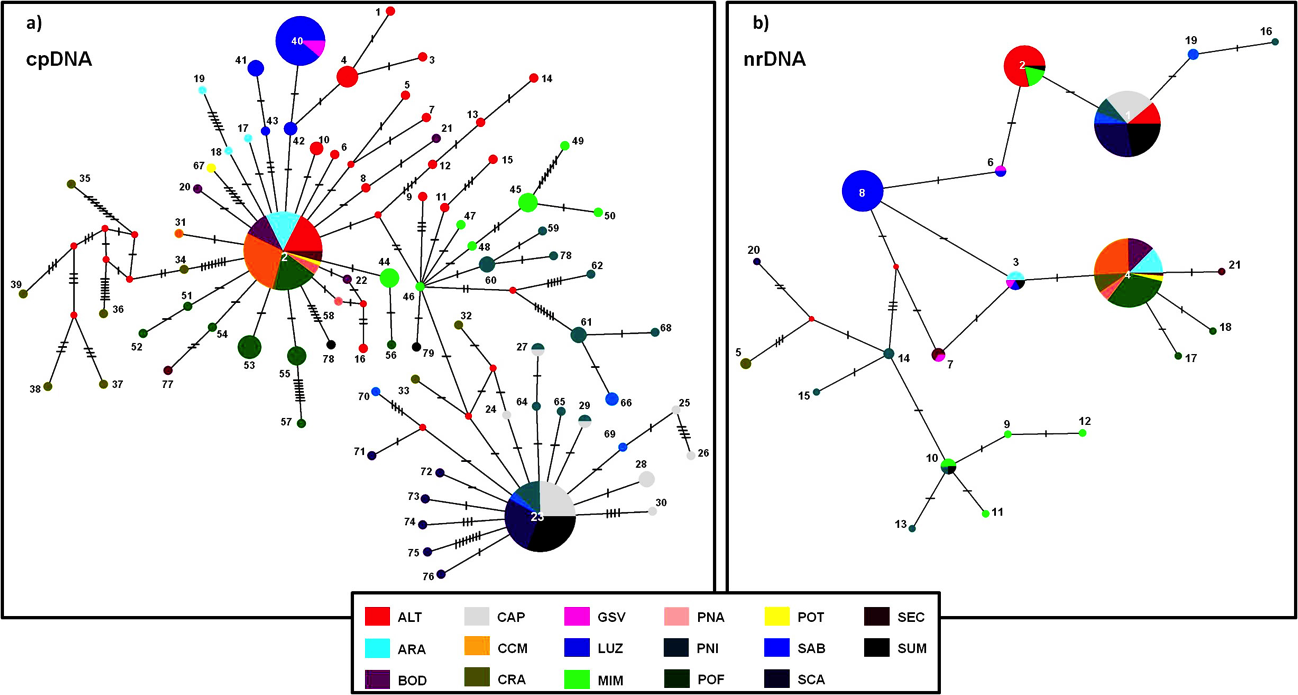


**Fig S3** Phylogenetic relationships among haplotypes of *Tabebuia serratifolia* for (**a**) cpDNA and (**b**) ITS nrDNA, using median-joining network. Circumference size is proportional to the haplotype frequency. Number of mutations is shown along lines in the network; small black circles are the median vectors. Different colours were assigned for each population according to the figure legend.


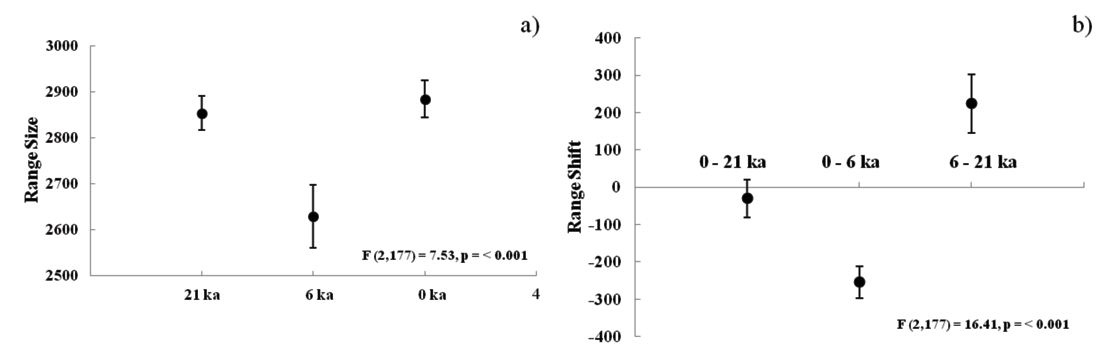


**Fig S4** Average and 95% confidence interval among the 60 maps of **(a)** range size and **(b)** shift (difference of range size among time periods in number of cells) predicted for *Tabebuia serratifolia* at LGM (21 ka), mid-Holocene (6 ka), and present-day (0 ka).


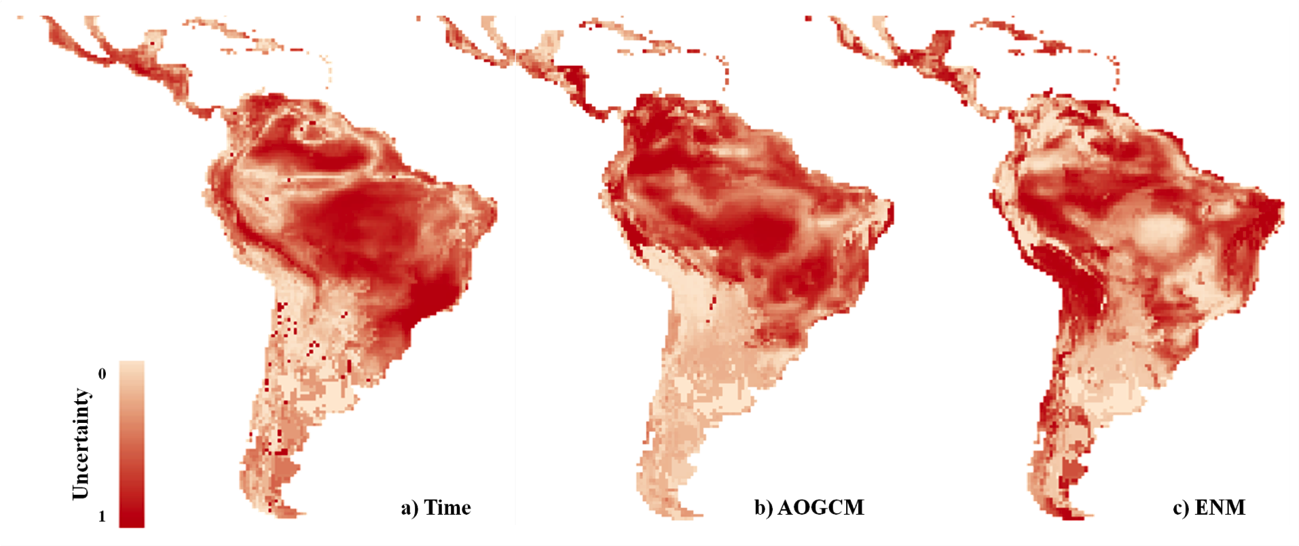


**Fig S5** Maps of uncertainty (relative sum of squares) for the modelling components of *Tabebuia serratifolia*, **(a)** Time, **(b)** Atmosphere-Ocean Global Circulation Models (AOGCMs), **(c)** Ecological Niche Models (ENMs).


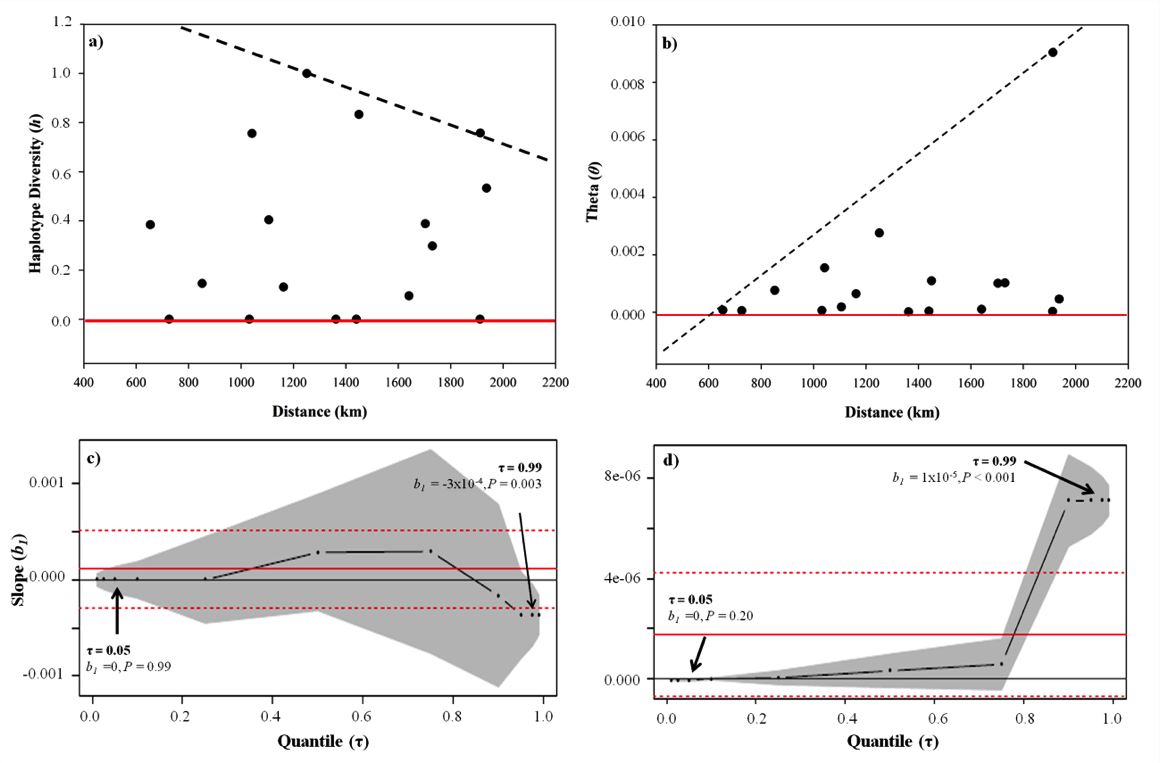


**Fig S6** Quantile regression for the relationships of haplotype diversity (*h*) (a) and Theta (*θ*) (b) among the 17 populations of *Tabebuia serratifolia* sampled in Brazil with distance from the centroid of distribution occupied by the species through the last glacial maximum. The upper figures (a, b) show the triangle-shaped envelopes from 0.05 (red line) and 0.99 (upper dashed line) quantile fits. The lower figures (c, d) show the respective slope estimates for 0.01, 0.025, 0.05, 0.1, 0.25, 0.5, 0.75, 0.9, 0.95, 0.975 and 0.99 quantiles (hatched area indicates 95% confidence interval around slope estimates).


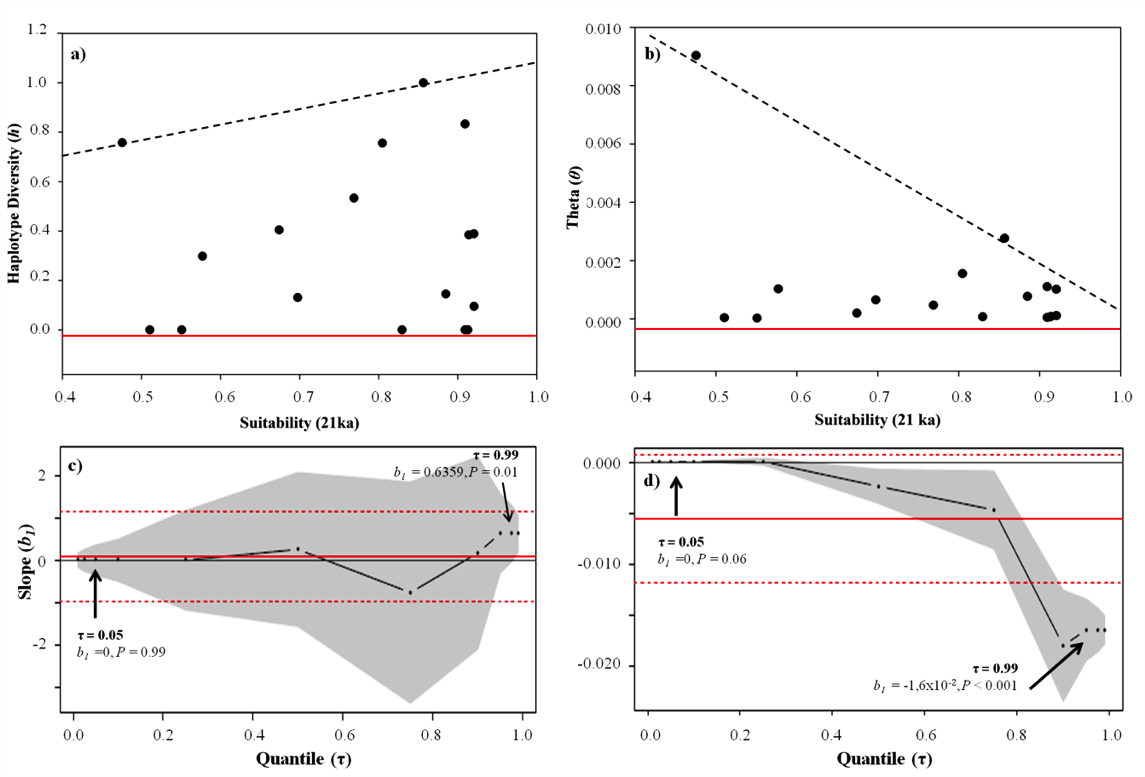


**Fig S7** Quantile regression for the relationships of haplotype diversity (*h*) (a) and Theta (*θ*) (b) among the 17 populations of *Tabebuia serratifolia* sampled in Brazil with suitability area presented by the species through the last glacial maximum. The upper figures (a, b) show the triangle-shaped envelopes from 0.05 (red line) and 0.99 (upper dashed line) quantile fits. The lower figures (c, d) show the respective slope estimates for 0.01, 0.025, 0.05, 0.1, 0.25, 0.5, 0.75, 0.9, 0.95, 0.975 and 0.99 quantiles (hatched area indicates 95% confidence interval around slope estimates).


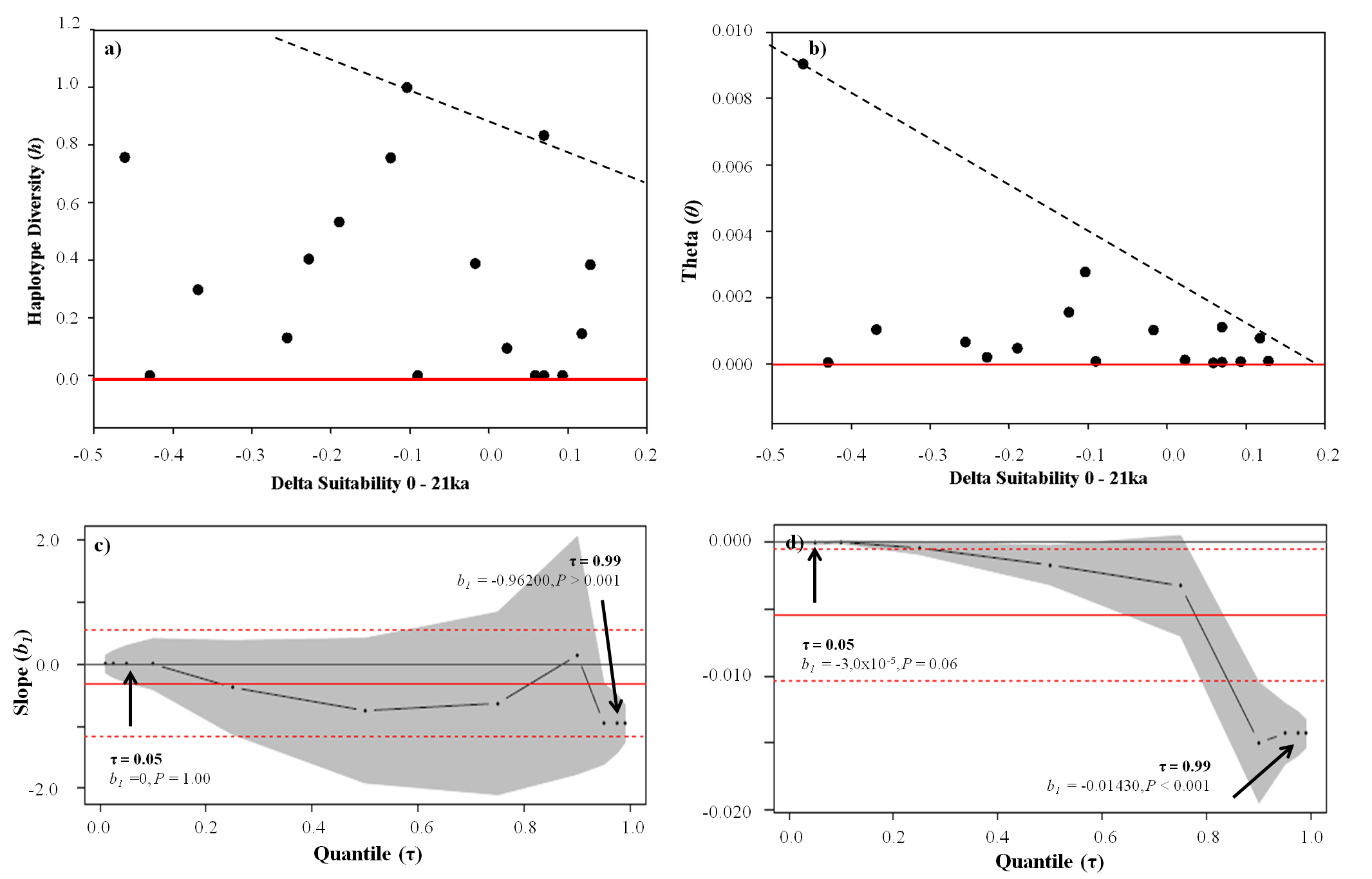


**Fig S8** Quantile regression for the relationships of haplotype diversity (*h*) (a) and Theta (*θ*) (b) among the 17 populations of *Tabebuia serratifolia* sampled in Brazil with difference in suitability through time. The upper figures (a, b) show the triangle-shaped envelopes from 0.05 (red line) and 0.99 (upper dashed line) quantile fits. The lower figures (c, d) show the respective slope estimates for 0.01, 0.025, 0.05, 0.1, 0.25, 0.5, 0.75, 0.9, 0.95, 0.975 and 0.99 quantiles (hatched area indicates 95% confidence interval around slope estimates).
