# Supplementary material for: Demographical history and palaeodistribution modelling show range shift towards Amazon Basin for a Neotropical tree species in the LGM
Source: BMC Evol Biol. 2016 Oct 13;16:213. doi: 10.1186/s12862-016-0779-9 (PMC5062830; doi:10.1186/s12862-016-0779-9)
Supplement: Additional file 2: Tables S1–S11. — with sampling locations and details on ecological niche modelling and demographical parameters. (DOCX 143 kb) [file 12862_2016_779_MOESM2_ESM.docx]

**Demographical history and palaeodistribution modelling show range shift towards Amazon Basin for a seasonally dry forest tree species in the LGM**

Luciana Cristina Vitorino, Matheus S. Lima-Ribeiro, Levi Carina Terribile, Rosane G. Collevatti

**Supporting Information - Tables**

**Table S1** Sampling location for the 17 populations of *Tabebuia serratifolia* from Brazil used for genetic analyses.

| **Population Locality** | | **Code** | | **Latitude** | | **Longitude** | |
| --- | --- | --- | --- | --- | --- | --- | --- |
| **North - Northeast** | |  | |  | |  | |
| Complexo Campo Maior, PI | | CCM | | S04 49 40.0 | | W 42 10 07.0 | |
| Crato, CE | | CRA | | S07 16 53.6 | | W39 19 20.4 | |
| Porto Franco, MA | | POF | | S06 19 57.2 | | W47 13 46.2 | |
| Sete Cidades, PI | | SEC | | S04 51 37.4 | | W42 03 36.5 | |
| **Central – Central West** | |  | |  | |  | |
| Parque Estadual Altamiro Pacheco, GO | | ALT | | S16 32 20.1 | | W49 08 17.5 | |
| Fazenda Araguaia, TO | | ARA | | S11 28 36.0 | | W49 46 33.3 | |
| Serra da Bodoquena, MS | | BOD | | S20 28 53.1 | | W55 31 24.4 | |
| Parque Nacional Grande Sertão Veredas, MG | | GSV | | S15 18 25.6 | | W45 48 12.0 | |
| Luziânia, GO | | LUZ | | S16 14 28.3 | | W47 54 06.9 | |
|  | |  | |  | |  | |
| Mimoso, GO | | MIM | | S15 02 15.5 | | W48 09 10.1 | |
| Portelândia, GO | | POT | | S17 17 45.5 | | W52 38 39.5 | |
| Porto Nacional, TO | | PNA | | S10 39 05.8 | | W48 35 53.6 | |
| **Southeast** | |  | |  | |  | |
| Cachoeira Paulista, SP | | CAP | | S22 40 01.0 | | W44 59 08.0 | |
| Parque Nacional de Itatiaia, RJ | | PNI | | S22 26 21.0 | | W44 36 28.0 | |
| São José do Barreiro, SP | | SAB | | S22 39 11.0 | | W44 34 56.0 | |
| Bambuí, Serra da Canastra, MG | | SCA | | S19 55 28.8 | | W45 55 36.0 | |
| Sumidouro, Lagoa Santa e Serra do Cipó, MG | | SUM | | S19 33 07.1 | | W43 55 55.8 | |
| Parque Estadual da Mata Seca, MG | *T. ochracea* | | S14 56 57.4 | | W44 04 10.7 | |  |
| Parque Estadual Altamiro Pacheco, GO | *T. impetiginosa* | | S16 32 20.1 | | W49 08 17.5 | |  |
| Sapucaia, RJ | *C. antisyphilitica* | | S21 58 28.7 | | W42 55 26.6 | |  |

**Table S2** Contemporary occurrence records (698) of *Tabebuia serratifolia* represented by the centroid of grid cells across the Neotropics used in the ecological niche modelling (ENM)**.**

| **Long** | **Lat** |  | **Long** | **Lat** |  | **Long** | **Lat** |  | **Long** | **Lat** |  | **Long** | **Lat** |
| --- | --- | --- | --- | --- | --- | --- | --- | --- | --- | --- | --- | --- | --- |
| -69.08 | -11.42 |  | -52.59 | -7.20 |  | -47.17 | -19.18 |  | -41.88 | -5.10 |  | -53.20 | 3.63 |
| -69.00 | -11.00 |  | -52.58 | -1.52 |  | -47.14 | -23.53 |  | -41.83 | -19.67 |  | -53.20 | 3.63 |
| -68.77 | -13.58 |  | -52.57 | -13.20 |  | -47.10 | -12.65 |  | -41.78 | -22.73 |  | -53.20 | 3.62 |
| -68.77 | -14.58 |  | -52.57 | -0.88 |  | -47.06 | -22.91 |  | -41.74 | -4.07 |  | -53.07 | 3.26 |
| -68.76 | -14.57 |  | -52.45 | 1.78 |  | -47.00 | -24.00 |  | -41.72 | -4.10 |  | -53.05 | 5.05 |
| -68.69 | -14.59 |  | -52.38 | -16.50 |  | -46.99 | -24.32 |  | -41.71 | -4.12 |  | -53.00 | 5.33 |
| -68.69 | -14.58 |  | -52.36 | -14.72 |  | -46.92 | -24.37 |  | -41.70 | -4.10 |  | -52.99 | 5.00 |
| -68.69 | -14.56 |  | -52.33 | -14.73 |  | -46.90 | -22.74 |  | -41.70 | -4.10 |  | -52.92 | 5.33 |
| -68.53 | -14.24 |  | -52.32 | 3.17 |  | -46.90 | -22.80 |  | -41.69 | -4.12 |  | -52.92 | 5.32 |
| -68.20 | -13.67 |  | -52.27 | -13.55 |  | -46.88 | -22.80 |  | -41.67 | -2.88 |  | -52.90 | 5.00 |
| -68.03 | -10.93 |  | -52.18 | -25.65 |  | -46.88 | -21.59 |  | -41.58 | -12.35 |  | -52.87 | 2.23 |
| -67.68 | -14.56 |  | -52.08 | 3.09 |  | -46.82 | -19.92 |  | -41.48 | -6.87 |  | -52.82 | 5.27 |
| -67.67 | -14.55 |  | -52.00 | 0.90 |  | -46.77 | -1.05 |  | -41.47 | -6.87 |  | -52.71 | 5.20 |
| -67.67 | -14.56 |  | -52.00 | 4.00 |  | -46.67 | -4.55 |  | -41.44 | -3.41 |  | -52.70 | 4.05 |
| -67.55 | -14.37 |  | -52.00 | -18.44 |  | -46.67 | -0.92 |  | -41.32 | -21.75 |  | -52.70 | 4.09 |
| -67.18 | -11.40 |  | -51.94 | -23.43 |  | -46.66 | -23.59 |  | -41.30 | -11.83 |  | -52.70 | 5.02 |
| -67.00 | -11.00 |  | -51.86 | 1.52 |  | -46.63 | -23.54 |  | -41.16 | -11.55 |  | -52.58 | 4.82 |
| -66.14 | -15.12 |  | -51.83 | -16.34 |  | -46.57 | -13.69 |  | -41.06 | -19.50 |  | -52.58 | 4.92 |
| -66.10 | -11.03 |  | -51.77 | -12.82 |  | -46.56 | -21.84 |  | -40.92 | -3.85 |  | -52.52 | 4.48 |
| -66.10 | -11.00 |  | -51.77 | -12.82 |  | -46.56 | -21.79 |  | -40.90 | -3.87 |  | -52.33 | 4.93 |
| -64.70 | -15.62 |  | -51.62 | 0.17 |  | -46.53 | -23.66 |  | -40.85 | -21.05 |  | -52.33 | 4.93 |
| -64.67 | -12.48 |  | -51.45 | 1.13 |  | -46.52 | -22.59 |  | -40.83 | -3.12 |  | -52.32 | 4.94 |
| -64.52 | -16.58 |  | -51.28 | 0.19 |  | -46.52 | -18.58 |  | -40.77 | -5.53 |  | -52.28 | 4.88 |
| -64.00 | -17.00 |  | -51.22 | -3.47 |  | -46.52 | -18.58 |  | -40.72 | -9.42 |  | -52.27 | 4.87 |
| -63.67 | -17.45 |  | -51.03 | 0.15 |  | -46.52 | -20.32 |  | -40.68 | -3.47 |  | -52.26 | 4.87 |
| -63.63 | -16.90 |  | -50.95 | -2.50 |  | -46.40 | -4.35 |  | -40.60 | -19.94 |  | -52.23 | 3.17 |
| -63.60 | -17.58 |  | -50.82 | -1.94 |  | -46.39 | -23.96 |  | -40.60 | -19.92 |  | -51.80 | 3.89 |
| -63.32 | -15.05 |  | -50.45 | -18.45 |  | -46.38 | -21.93 |  | -40.57 | -19.84 |  | -76.22 | -5.98 |
| -62.75 | -14.75 |  | -50.45 | -16.95 |  | -46.38 | -21.93 |  | -40.53 | -20.27 |  | -75.48 | -8.72 |
| -62.62 | -14.63 |  | -50.27 | -6.05 |  | -46.37 | -21.33 |  | -40.53 | -19.97 |  | -75.17 | -8.67 |
| -62.60 | -14.67 |  | -50.20 | -9.82 |  | -46.37 | -21.33 |  | -40.50 | -20.04 |  | -75.02 | -8.75 |
| -61.85 | -16.52 |  | -50.15 | 1.11 |  | -46.35 | -20.27 |  | -40.47 | -20.06 |  | -75.00 | -8.67 |
| -61.80 | -15.06 |  | -50.11 | -6.05 |  | -46.32 | -22.85 |  | -40.47 | -9.37 |  | -74.96 | -8.65 |
| -61.55 | -13.60 |  | -50.09 | -20.51 |  | -46.20 | -21.51 |  | -40.31 | -20.13 |  | -74.95 | -9.30 |
| -61.50 | -17.50 |  | -50.04 | -23.52 |  | -46.19 | -23.52 |  | -40.27 | -19.82 |  | -74.58 | -8.42 |
| -61.16 | -14.72 |  | -50.04 | -23.52 |  | -46.00 | -24.00 |  | -40.27 | -20.10 |  | -74.57 | -8.38 |
| -61.15 | -14.39 |  | -50.00 | -10.00 |  | -46.00 | -23.58 |  | -40.15 | -19.36 |  | -74.57 | -8.35 |
| -61.14 | -14.71 |  | -49.88 | -6.07 |  | -45.96 | -14.03 |  | -40.07 | -19.39 |  | -74.14 | -9.18 |
| -61.14 | -14.75 |  | -49.62 | -10.79 |  | -45.90 | -1.97 |  | -40.07 | -18.75 |  | -73.00 | -11.68 |
| -61.13 | -15.01 |  | -49.50 | -15.50 |  | -45.70 | -1.24 |  | -40.05 | -19.23 |  | -72.97 | -3.50 |
| -60.90 | -13.58 |  | -49.46 | -4.01 |  | -45.59 | -22.73 |  | -40.01 | -19.02 |  | -72.92 | -3.25 |
| -60.81 | -13.93 |  | -49.38 | -20.82 |  | -45.57 | -4.05 |  | -40.00 | -19.17 |  | -72.87 | -11.78 |
| -60.75 | -14.56 |  | -49.34 | -14.20 |  | -45.52 | -23.64 |  | -39.99 | -14.84 |  | -72.83 | -3.47 |
| -60.74 | -14.52 |  | -49.33 | -15.75 |  | -45.47 | -22.68 |  | -39.88 | -14.90 |  | -71.72 | -12.12 |
| -59.70 | -17.30 |  | -49.17 | -12.20 |  | -45.45 | -1.85 |  | -39.88 | -19.67 |  | -71.30 | -11.92 |
| -59.68 | -17.34 |  | -49.15 | -14.46 |  | -45.41 | -23.76 |  | -39.83 | -18.40 |  | -71.25 | -12.58 |
| -58.18 | -18.65 |  | -49.13 | -15.78 |  | -45.38 | -3.67 |  | -39.72 | -7.51 |  | -71.25 | -12.17 |
| -58.00 | -19.00 |  | -49.12 | -5.37 |  | -45.37 | -1.66 |  | -39.65 | -7.12 |  | -71.17 | -11.75 |
| -73.65 | -7.48 |  | -49.10 | -15.30 |  | -45.35 | -23.00 |  | -39.64 | -13.72 |  | -71.00 | -11.75 |
| -73.15 | -7.67 |  | -49.10 | -17.73 |  | -45.33 | -15.37 |  | -39.55 | -14.32 |  | -70.25 | -12.00 |
| -71.90 | -9.38 |  | -49.06 | -17.82 |  | -45.31 | -23.22 |  | -39.50 | -7.45 |  | -70.10 | -12.57 |
| -70.03 | -4.38 |  | -49.06 | -22.31 |  | -45.27 | -18.21 |  | -39.44 | -7.28 |  | -69.39 | -12.69 |
| -69.00 | -9.33 |  | -48.99 | -14.52 |  | -45.25 | -3.49 |  | -39.41 | -7.23 |  | -69.28 | -12.83 |
| -68.75 | -11.02 |  | -48.99 | -14.62 |  | -45.19 | -11.05 |  | -39.39 | -7.32 |  | -69.21 | -12.61 |
| -68.66 | -9.07 |  | -48.95 | -2.95 |  | -45.19 | -22.81 |  | -39.30 | -7.31 |  | -69.05 | -12.58 |
| -68.66 | -9.07 |  | -48.84 | -14.50 |  | -45.12 | -22.73 |  | -39.28 | -14.79 |  | -69.05 | -12.48 |
| -68.15 | 0.58 |  | -48.83 | -25.48 |  | -45.08 | -23.37 |  | -39.19 | -13.57 |  | -56.50 | 4.00 |
| -67.68 | -8.27 |  | -48.56 | -16.12 |  | -45.00 | -21.25 |  | -39.18 | -16.38 |  | -55.63 | 4.00 |
| -65.83 | -8.92 |  | -48.54 | -20.71 |  | -44.97 | -4.43 |  | -39.15 | -16.37 |  | -55.22 | 5.70 |
| -65.69 | -8.89 |  | -48.51 | -16.25 |  | -44.97 | -21.22 |  | -39.13 | -7.40 |  | -55.05 | 5.14 |
| -65.29 | -10.66 |  | -48.51 | -25.52 |  | -44.92 | -5.42 |  | -39.08 | -12.53 |  | -55.00 | 6.00 |
| -64.83 | -9.54 |  | -48.51 | -0.67 |  | -44.92 | -5.42 |  | -39.08 | -15.15 |  | -55.00 | 5.00 |
| -64.40 | -9.25 |  | -48.50 | -1.46 |  | -44.90 | -2.52 |  | -39.08 | -14.78 |  | -61.50 | 10.67 |
| -64.39 | -9.27 |  | -48.48 | -15.87 |  | -44.90 | -18.76 |  | -39.08 | -15.29 |  | -61.00 | 10.28 |
| -64.23 | -12.45 |  | -48.48 | -1.40 |  | -44.89 | -21.96 |  | -39.06 | -16.45 |  | -72.07 | 7.58 |
| -64.13 | -3.21 |  | -48.46 | -14.47 |  | -44.87 | -18.83 |  | -38.97 | -4.21 |  | -72.05 | 7.52 |
| -63.90 | -8.76 |  | -48.42 | -13.82 |  | -44.71 | -23.22 |  | -38.96 | -12.62 |  | -71.95 | 7.35 |
| -63.88 | -8.77 |  | -48.37 | -9.87 |  | -44.68 | -22.50 |  | -38.93 | -4.20 |  | -71.93 | 7.40 |
| -63.61 | -10.60 |  | -48.36 | -10.21 |  | -44.66 | -22.36 |  | -38.90 | -4.47 |  | -71.93 | 7.15 |
| -63.32 | 0.82 |  | -48.35 | -9.75 |  | -44.64 | -20.54 |  | -38.87 | -3.88 |  | -71.48 | 7.75 |
| -63.10 | -2.28 |  | -48.32 | -21.25 |  | -44.57 | -22.25 |  | -38.69 | -3.89 |  | -71.42 | 7.17 |
| -62.96 | -9.23 |  | -48.30 | -10.43 |  | -44.56 | -22.50 |  | -38.65 | -3.74 |  | -71.30 | 7.68 |
| -62.42 | 3.22 |  | -48.28 | -18.92 |  | -44.56 | -7.23 |  | -38.30 | -7.98 |  | -71.28 | 7.90 |
| -61.70 | 3.50 |  | -48.23 | -12.82 |  | -44.46 | -4.88 |  | -38.15 | -7.82 |  | -71.02 | 10.40 |
| -61.43 | 3.37 |  | -48.22 | -16.31 |  | -44.40 | -19.27 |  | -38.02 | -8.66 |  | -70.97 | 10.42 |
| -61.32 | -11.82 |  | -48.22 | -12.74 |  | -44.40 | -19.27 |  | -37.72 | -12.04 |  | -70.39 | 9.77 |
| -61.25 | 1.52 |  | -48.21 | -12.49 |  | -44.38 | -5.56 |  | -37.69 | -9.81 |  | -70.10 | 8.63 |
| -60.90 | -12.55 |  | -48.20 | -1.96 |  | -44.32 | -23.01 |  | -37.68 | -9.81 |  | -69.20 | 9.56 |
| -60.89 | -12.96 |  | -48.19 | -18.65 |  | -44.31 | -10.04 |  | -37.61 | -11.81 |  | -68.92 | 10.12 |
| -60.68 | 2.84 |  | -48.17 | -15.70 |  | -44.30 | -2.53 |  | -37.40 | -6.67 |  | -68.62 | 9.63 |
| -60.67 | 2.82 |  | -48.13 | -18.77 |  | -44.27 | -20.05 |  | -36.91 | -5.58 |  | -68.19 | 10.12 |
| -60.55 | -13.49 |  | -48.06 | -16.02 |  | -44.25 | -2.55 |  | -35.85 | -7.17 |  | -67.68 | 5.38 |
| -60.54 | -13.12 |  | -48.05 | -15.55 |  | -44.25 | -6.03 |  | -35.73 | -8.47 |  | -67.62 | 5.66 |
| -60.34 | -14.00 |  | -48.05 | -15.87 |  | -44.25 | -19.47 |  | -35.69 | -6.96 |  | -67.60 | 5.63 |
| -60.08 | -2.32 |  | -48.02 | -16.64 |  | -44.25 | -19.47 |  | -35.63 | -7.16 |  | -67.58 | 5.58 |
| -60.05 | -3.02 |  | -48.01 | -15.91 |  | -44.21 | -14.23 |  | -35.46 | -8.24 |  | -67.17 | 6.35 |
| -60.03 | -3.10 |  | -48.00 | -25.22 |  | -44.21 | -2.96 |  | -35.37 | -7.52 |  | -67.00 | 5.00 |
| -60.03 | -2.03 |  | -47.99 | -24.06 |  | -44.20 | -19.97 |  | -35.33 | -7.66 |  | -66.95 | 10.48 |
| -59.97 | -2.88 |  | -47.98 | -15.67 |  | -44.18 | -22.00 |  | -35.16 | -6.73 |  | -66.82 | 10.43 |
| -59.97 | -2.88 |  | -47.96 | -21.32 |  | -44.09 | -19.77 |  | -35.08 | -6.18 |  | -66.82 | 1.22 |
| -59.95 | -2.37 |  | -47.95 | -16.25 |  | -44.08 | -18.64 |  | -35.02 | -8.00 |  | -66.80 | 10.45 |
| -59.92 | -2.96 |  | -47.95 | -16.25 |  | -44.08 | -19.56 |  | -35.02 | -8.00 |  | -66.78 | 10.08 |
| -59.90 | -2.14 |  | -47.94 | -21.34 |  | -44.00 | -18.00 |  | -34.95 | -8.01 |  | -66.50 | 6.92 |
| -59.85 | -2.38 |  | -47.93 | -15.78 |  | -44.00 | -19.85 |  | -34.86 | -7.12 |  | -66.42 | 10.08 |
| -59.85 | -2.42 |  | -47.93 | -1.29 |  | -43.99 | -19.47 |  | -75.50 | 0.45 |  | -66.25 | 0.87 |
| -59.85 | -2.38 |  | -47.92 | -24.18 |  | -43.97 | -19.53 |  | -75.34 | 9.54 |  | -66.23 | 6.65 |
| -59.80 | -2.43 |  | -47.92 | -15.78 |  | -43.95 | -19.85 |  | -75.23 | 8.08 |  | -66.02 | 4.05 |
| -59.77 | -2.73 |  | -47.92 | -25.01 |  | -43.95 | -19.73 |  | -75.14 | 8.05 |  | -66.02 | 10.08 |
| -59.70 | -2.70 |  | -47.92 | -24.38 |  | -43.95 | -17.28 |  | -74.87 | 10.68 |  | -66.02 | 5.32 |
| -59.61 | -10.28 |  | -47.92 | -15.78 |  | -43.94 | -19.92 |  | -74.63 | 5.53 |  | -65.80 | 10.15 |
| -59.52 | -14.29 |  | -47.90 | -16.22 |  | -43.89 | -19.63 |  | -73.85 | 7.07 |  | -65.73 | 3.73 |
| -59.46 | -10.17 |  | -47.89 | -15.74 |  | -43.89 | -19.63 |  | -73.85 | 0.87 |  | -65.32 | 10.05 |
| -59.00 | -2.00 |  | -47.88 | -15.58 |  | -43.86 | -16.74 |  | -73.85 | 7.07 |  | -65.18 | 7.28 |
| -58.78 | -10.37 |  | -47.87 | -24.20 |  | -43.85 | -19.77 |  | -73.77 | 7.06 |  | -65.17 | 7.33 |
| -58.47 | -1.92 |  | -47.87 | -15.75 |  | -43.85 | -19.99 |  | -73.75 | 8.33 |  | -64.68 | 5.47 |
| -58.44 | -3.14 |  | -47.87 | -8.83 |  | -43.57 | -6.79 |  | -72.63 | 6.65 |  | -64.60 | 10.23 |
| -57.68 | -16.07 |  | -47.83 | -8.44 |  | -43.53 | -22.67 |  | -73.39 | 4.15 |  | -64.50 | 6.32 |
| -57.57 | -11.73 |  | -47.83 | -21.10 |  | -43.43 | -22.97 |  | -73.55 | 7.38 |  | -64.37 | 10.00 |
| -57.41 | -11.54 |  | -47.82 | -15.85 |  | -43.43 | -9.25 |  | -72.64 | 0.52 |  | -64.33 | 10.33 |
| -57.31 | -5.73 |  | -47.81 | -21.17 |  | -43.40 | -3.21 |  | -71.59 | 1.05 |  | -64.32 | 10.33 |
| -57.00 | -12.00 |  | -47.81 | -21.09 |  | -43.39 | -17.80 |  | -70.24 | 5.66 |  | -64.25 | 8.90 |
| -56.61 | -4.71 |  | -47.80 | -21.30 |  | -43.28 | -22.93 |  | -70.00 | -3.03 |  | -63.57 | 6.33 |
| -56.58 | -16.33 |  | -47.76 | -21.13 |  | -43.27 | -22.70 |  | -69.95 | -4.15 |  | -63.50 | 10.62 |
| -56.48 | -9.66 |  | -47.75 | -7.14 |  | -43.27 | -21.59 |  | -69.94 | 4.22 |  | -63.12 | 6.95 |
| -56.18 | -3.22 |  | -47.75 | -21.13 |  | -43.21 | -22.90 |  | -69.85 | -1.87 |  | -62.97 | 7.58 |
| -56.08 | -1.37 |  | -47.74 | -21.15 |  | -43.21 | -22.90 |  | -67.88 | 3.83 |  | -62.25 | 10.65 |
| -56.02 | -9.92 |  | -47.73 | -15.82 |  | -43.17 | -19.81 |  | -67.67 | 5.67 |  | -61.73 | 8.67 |
| -56.01 | -9.74 |  | -47.73 | -16.98 |  | -43.14 | -7.78 |  | -67.64 | 5.68 |  | -61.73 | 8.23 |
| -56.00 | -15.00 |  | -47.73 | -21.13 |  | -43.10 | -22.88 |  | -77.68 | -2.85 |  | -61.73 | 8.23 |
| -56.00 | -16.00 |  | -47.72 | -21.16 |  | -43.10 | -22.90 |  | -77.52 | -1.07 |  | -61.63 | 6.72 |
| -55.97 | -16.19 |  | -47.71 | -7.30 |  | -42.87 | -20.75 |  | -77.30 | -1.19 |  | -61.38 | 7.07 |
| -55.97 | -9.90 |  | -47.70 | -18.04 |  | -42.85 | -6.24 |  | -77.21 | -2.26 |  | -49.14 | -16.54 |
| -55.92 | -9.59 |  | -47.68 | -11.87 |  | -42.84 | -5.09 |  | -77.00 | -0.47 |  | -49.78 | -11.48 |
| -55.87 | -1.77 |  | -47.67 | -21.13 |  | -42.70 | -9.02 |  | -76.46 | -0.63 |  | -55.52 | -20.48 |
| -55.75 | -15.46 |  | -47.67 | -8.43 |  | -42.61 | -5.56 |  | -76.45 | -0.62 |  | -44.99 | -22.67 |
| -55.52 | -2.00 |  | -47.64 | -15.76 |  | -42.50 | -19.52 |  | -75.80 | -1.10 |  | -42.17 | -4.83 |
| -55.50 | -11.86 |  | -47.64 | -22.72 |  | -42.50 | -22.05 |  | -59.70 | 7.37 |  | -39.32 | -7.28 |
| -55.49 | -3.83 |  | -47.62 | -22.70 |  | -42.40 | -13.84 |  | -59.33 | 2.42 |  | -45.80 | -15.31 |
| -55.21 | -9.67 |  | -47.61 | -15.56 |  | -42.25 | -22.50 |  | -59.33 | 2.42 |  | -47.90 | -16.24 |
| -55.02 | -2.92 |  | -47.55 | -24.70 |  | -42.25 | -13.30 |  | -59.25 | 2.42 |  | -48.15 | -15.04 |
| -54.99 | -19.39 |  | -47.49 | -15.98 |  | -42.14 | -19.79 |  | -59.03 | 2.93 |  | -48.60 | -10.65 |
| -54.94 | -2.64 |  | -47.49 | -7.35 |  | -42.13 | -20.23 |  | -58.97 | 2.91 |  | -44.61 | -22.44 |
| -54.71 | -2.44 |  | -47.48 | -5.55 |  | -42.13 | -7.03 |  | -54.38 | 4.28 |  | -47.23 | -6.33 |
| -54.64 | -16.47 |  | -47.48 | -5.53 |  | -42.10 | -22.84 |  | -54.10 | 3.25 |  | -52.64 | -17.30 |
| -54.07 | -2.01 |  | -47.46 | -24.28 |  | -42.03 | -20.77 |  | -54.02 | 3.42 |  | -44.58 | -22.65 |
| -53.38 | -13.10 |  | -47.33 | -15.54 |  | -42.02 | -22.88 |  | -54.02 | 3.42 |  | -45.93 | -9.93 |
| -53.19 | -0.38 |  | -47.29 | -19.35 |  | -42.00 | -22.62 |  | -54.02 | 5.40 |  | -42.06 | -4.86 |
| -53.00 | 2.00 |  | -47.29 | -22.77 |  | -41.97 | -22.80 |  | -53.95 | 4.74 |  | -43.93 | -19.55 |
| -52.71 | 2.36 |  | -47.25 | -15.33 |  | -41.97 | -22.82 |  | -53.88 | 5.62 |  |  |  |
| -52.67 | -16.45 |  | -47.18 | -1.20 |  | -41.92 | -22.77 |  | -53.22 | 3.62 |  |  |  |

**Table S3** Details on the palaeoclimatic simulations (AOGCMs) used in the ecological niche modelling of *Tabebuia serratifolia.*

| **Model ID** | **Modeling Center** | **Resolution*** | **Source** | **Year** |
| --- | --- | --- | --- | --- |
| CCSM4 | University of Miami – RSMAS. USA | 0.9° × 1.25° | CMIP5/PMIP3 | 2012 |
| CNRM-CM5 | Centre National de Recherches Meteorologiques / Centre Europeen de Recherche et Formation Avancees en Calcul Scientifique. France | 1.4° x 1.4° | CMIP5/PMIP3 | 2012 |
| MIROC-ESM | Atmosphere and Ocean Research Institute (University of Tokyo). National Institute for Environmental Studies. and Japan Agency for Marine-Earth Science and Technology. Japan | 2.8° × 2.8° | CMIP5/PMIP3 | 2012 |
| MPI-ESM-P | Max Planck Institute for Meteorology, Germany | 1.9° × 1.9° | CMIP5/PMIP3 | 2011 |
| MRI-CGCM3 | Meteorological Research Institute. Japan | 1.1° x 1.1° | CMIP5/PMIP3 | 2012 |

*longitude × latitude

CMIP5 – Coupled Model Intercomparison Project. Phase 5 (<http://cmip-pcmdi.llnl.gov/>)

PMIP3 – Paleoclimate Modelling Intercomparison Project. Phase 3 (<http://pmip3.lsce.ipsl.fr/>)

**Table S4** Ecological niche modelling methods used to estimate *Tabebuia serratifolia* potential distribution.

| **Method** | **Species data type** |
| --- | --- |
| Bioclimatic Envelope (BIOCLIM) | Presence only |
| Ecological Niche Factor Analysis (ENFA) | Presence only |
| Euclidian Distance (EuclidDist) | Presence only |
| Generalized Linear Models (GLM) | Presence and absence |
| Gower Distance (GowerDist) | Presence only |
| Mahalanobis Distance (MahalDist) | Presence only |
| Maximum Entropy (Maxent) | Presence/background |
| Generalized additive models (GAM) | Presence and absence |
| Flexible discriminant analysis (FDA) | Presence and absence |
| Multivariate adaptive regression splines (MARS) | Presence and absence |
| Neural Networks (ANN) | Presence and absence |
| Random Forest (RNDFOR) | Presence and absence |

**Table S5** TSS and AUC values for ENMs and AOGCMs models used to model the palaeodistribution of *Tabebuia serratifolia* in Neotropical SDFs.

|  |  |  |  | **AOGCMs** |  |  |  |  |
| --- | --- | --- | --- | --- | --- | --- | --- | --- |
|  |  | **CCSM** | **CNRM** | **MIROC** | **MPI** | **MRI** | **Mean** | **SD** |
| **ENM Algorithms - TSS** | **BioClim** | 0.705 | 0.321 | 0.318 | 0.297 | 0.314 | 0.391 | 0.176 |
|  | **ENFA** | 0.311 | 0.323 | 0.350 | 0.321 | 0.351 | 0.331 | 0.018 |
|  | **EuclidDist** | 0.268 | 0.257 | 0.326 | 0.289 | 0.251 | 0.278 | 0.030 |
|  | **FDA** | 0.358 | 0.349 | 0.346 | 0.360 | 0.330 | 0.349 | 0.012 |
|  | **GAM** | 0.371 | 0.378 | 0.370 | 0.436 | 0.322 | 0.375 | 0.041 |
|  | **GLM** | 0.356 | 0.369 | 0.358 | 0.408 | 0.327 | 0.363 | 0.029 |
|  | **GowerDist** | 0.291 | 0.261 | 0.308 | 0.276 | 0.274 | 0.282 | 0.018 |
|  | **MahalanobisDist** | 0.364 | 0.283 | 0.337 | 0.310 | 0.260 | 0.311 | 0.041 |
|  | **MARS** | 0.367 | 0.370 | 0.319 | 0.411 | 0.318 | 0.357 | 0.039 |
|  | **Maxent** | 0.348 | 0.368 | 0.358 | 0.453 | 0.314 | 0.368 | 0.052 |
|  | **Nnet** | 0.301 | 0.313 | 0.280 | 0.365 | 0.337 | 0.319 | 0.033 |
|  | **RndFor** | 0.403 | 0.394 | 0.431 | 0.397 | 0.382 | 0.402 | 0.018 |
|  | **Mean** | 0.370 | 0.332 | 0.342 | 0.360 | 0.315 |  |  |
|  | **SD** | 0.113 | 0.047 | 0.038 | 0.061 | 0.038 |  |  |
| **ENM Algorithms - AUC** | **BioClim** | 0.262 | 0.736 | 0.722 | 0.734 | 0.704 | 0.632 | 0.207 |
|  | **ENFA** | 0.694 | 0.722 | 0.720 | 0.708 | 0.708 | 0.710 | 0.011 |
|  | **EuclidDist** | 0.670 | 0.684 | 0.697 | 0.709 | 0.669 | 0.686 | 0.018 |
|  | **FDA** | 0.795 | 0.786 | 0.780 | 0.791 | 0.788 | 0.788 | 0.006 |
|  | **GAM** | 0.790 | 0.804 | 0.804 | 0.828 | 0.795 | 0.804 | 0.015 |
|  | **GLM** | 0.783 | 0.782 | 0.776 | 0.797 | 0.758 | 0.779 | 0.014 |
|  | **GowerDist** | 0.668 | 0.685 | 0.697 | 0.706 | 0.682 | 0.687 | 0.015 |
|  | **MahalanobisDist** | 0.710 | 0.685 | 0.716 | 0.713 | 0.684 | 0.702 | 0.016 |
|  | **MARS** | 0.784 | 0.799 | 0.791 | 0.826 | 0.784 | 0.797 | 0.017 |
|  | **Maxent** | 0.832 | 0.832 | 0.836 | 0.853 | 0.816 | 0.834 | 0.013 |
|  | **Nnet** | 0.786 | 0.792 | 0.803 | 0.833 | 0.820 | 0.807 | 0.020 |
|  | **RndFor** | 1.000 | 1.000 | 1.000 | 1.000 | 1.000 | 1.000 | 0.000 |
|  | **Mean** | 0.731 | 0.775 | 0.779 | 0.791 | 0.767 |  |  |
|  | **SD** | 0.173 | 0.088 | 0.084 | 0.086 | 0.092 |  |  |

**Table S6** Pairwise *φ_ST_* among the 17 populations of *Tabebuia serratifolia* sampled in Brazil. Nuclear ITS below diagonal and chloroplast DNA above diagonal. Values in bold are significant, *p* < 0.05.

|  | **CCM** |  | **CRA** |  | **POF** |  | **SEC** |  | **ALT** | **ARA** | **BOD** | **GSV** |  | **LUZ** |  | **MIM** |  | **POT** |  | **PNA** |  | **CAP** | **PNI** | **SAB** | **SCA** | **SUM** |
| --- | --- | --- | --- | --- | --- | --- | --- | --- | --- | --- | --- | --- | --- | --- | --- | --- | --- | --- | --- | --- | --- | --- | --- | --- | --- | --- |
| **CCM** |  |  | **0.562** |  | **0.574** |  | 0.335 |  | **0.943** | 0.227 | 0.000 | **0.956** |  | **0.646** |  | **0.524** |  | 0.785 |  | **0.070** |  | **1.000** | 0.213 | **0.741** | **0.850** | **0.841** |
| **CRA** | 0.258 |  |  |  | **0.462** |  | **0.276** |  | **0.750** | 0.134 | 0.141 | **0.340** |  | **0.631** |  | **0.416** |  | 0.223 |  | **0.516** |  | **0.765** | **0.278** | **0.390** | **0.633** | **0.604** |
| **POF** | -0.012 |  | 0.290 |  |  |  | **0.409** |  | **0.935** | **0.163** | 00.045 | **0.821** |  | **0.932** |  | **0.740** |  | 0.430 |  | 0.140 |  | **0.982** | **0.762** | 0.008 | **0.186** | **0.146** |
| **SEC** | **0.677** |  | 0.003 |  | **0.617** |  |  |  | **0.850** | 0.290 | **0.513** | 0.091 |  | **0.831** |  | **0.329** |  | 0.033 |  | 0.266 |  | **0.936** | **0.409** | **0.780** | **0.873** | **0.286** |
| **ALT** | **0.059** |  | **0.447** |  | **0.423** |  | 0.019 |  |  | 0.041 | 0.021 | **0.248** |  | **0.351** |  | **0.316** |  | 0.201 |  | **0.087** |  | **0.607** | -0.060 | **0.487** | **0.602** | **0.558** |
| **ARA** | 0.049 |  | **0.441** |  | **0.492** |  | 0.018 |  | **0.901** |  | 0.005 | **0.610** |  | **0.541** |  | **0.405** |  | 0.420 |  | **0.053** |  | **0.767** | -0.069 | **0.595** | **0.766** | **0.743** |
| **BOD** | 0.052 |  | **0.416** |  | **0.481** |  | 0.055 |  | **0.929** | 0.133 |  | **0.746** |  | **0.560** |  | **0.395** |  | 0.490 |  | 0.033 |  | **0.783** | -0.034 | **0.582** | **0.785** | **0.765** |
| **GSV** | **0.872** |  | 0.080 |  | **0.500** |  | **0.681** |  | **0.796** | **0.565** | **0.775** |  |  | 0.119 |  | **0.439** |  | 0.543 |  | **0.489** |  | **0.933** | **0.868** | **0.500** | **0.821** | **0.811** |
| **LUZ** | **0.956** |  | **0.611** |  | **0.642** |  | **0.538** |  | **0.881** | **0.880** | **0.950** | **0.686** |  |  |  | **0.610** |  | **0.654** |  | **0.504** |  | **0.972** | **0.538** | **0.737** | **0.817** | **0.802** |
| **MIM** | **0.712** |  | **0.326** |  | **0.268** |  | **0.296** |  | **0.519** | **0.591** | **0.606** | 0.179 |  | **0.586** |  |  |  | **0.329** |  | **0.438** |  | **0.511** | **0.295** | **0.316** | **0.513** | **0.464** |
| **POT** | 0.000 |  | 0.194 |  | 0.332 |  | 0.175 |  | **0.910** | -0.161 | 0.000 | 0.368 |  | **0.936** |  | **0.380** |  |  |  | 0.000 |  | **1.000** | **0.461** | 0.355 | **0.770** | **0.751** |
| **PNA** | 0.000 |  | 0.005 |  | **0.531** |  | 0.014 |  | **0.916** | 0.010 | 0.000 | **0.579** |  | **0.941** |  | **0.478** |  | 0.371 |  |  |  | **1.000** | -0.036 | **0.630** | **0.734** | **0.705** |
| **CAP** | **0.846** |  | **0.684** |  | **0.187** |  | **0.757** |  | **0.674** | **0.966** | **1.000** | **0.821** |  | **0.817** |  | **0.521** |  | **0.773** |  | **0.736** |  |  | **0.768** | **0.310** | 0.012 | **0.019** |
| **PNI** | **0.737** |  | **0.382** |  | **0.408** |  | 0.000 |  | **0.508** | **0.634** | **0.645** | **0.283** |  | **0.645** |  | 0.022 |  | 0.323 |  | **0.540** |  | **0.358** |  | **0.446** | **0.768** | **0.748** |
| **SAB** | **0.979** |  | **0.591** |  | **0.960** |  | **0.432** |  | **0.589** | **0.916** | **0.964** | **0.735** |  | **0.940** |  | 0.268 |  | **0.918** |  | **0.938** |  | 0.446 | 0.123 |  | **0.311** | **0.286** |
| **SCA** | **0.962** |  | **0.738** |  | **0.952** |  | **0.757** |  | **0.573** | **0.921** | **0.950** | **0.843** |  | **0.923** |  | **0.476** |  | **0.930** |  | **0.936** |  | -0.008 | **0.315** | 0.156 |  | 0.432 |
| **SUM** | **0.887** |  | **0.626** |  | **0.887** |  | **0.722** |  | **0.371** | **0.823** | **0.850** | **0.639** |  | **0.820** |  | **0.335** |  | **0.791** |  | **0.812** |  | 0.028 | **0.204** | 0.014 | -0.001 |  |

**Table S7** Number of migrants per generation (*N_e_m* ) for the 17 populations of *Tabebuia serratifolia* in Brazil, based on Bayesian coalescent analysis. Migration direction is from populations in the rows into populations in the columns. Note that all values of *N_e_m* are < 1.00.

|  | **CCM** | **CRA** | **POF** | **ALT** | **ARA** | **BOD** | **LUZ** | **MIM** | **CAP** | **PNI** | **SAB** | **SCA** | **SUM** |
| --- | --- | --- | --- | --- | --- | --- | --- | --- | --- | --- | --- | --- | --- |
| **CCM** |  | 0.604 | 0.077 | 0.647 | 0.057 | 0.023 | 0.045 | 0.026 | 0.026 | 0.048 | 0.051 | 0.009 | 0.009 |
| **CRA** | 0.003 |  | 0.017 | 0.089 | 0.018 | 0.005 | 0.020 | 0.021 | 0.014 | 0.032 | 0.031 | 0.043 | 0.005 |
| **POF** | 0.006 | 0.484 |  | 0.085 | 0.041 | 0.017 | 0.013 | 0.051 | 0.011 | 0.068 | 0.015 | 0.011 | 0.010 |
| **ALT** | 0.004 | 0.078 | 0.040 |  | 0.009 | 0.012 | 0.014 | 0.212 | 0.015 | 0.097 | 0.021 | 0.021 | 0.047 |
| **ARA** | 0.005 | 0.138 | 0.017 | 0.584 |  | 0.023 | 0.054 | 0.022 | 0.008 | 0.046 | 0.029 | 0.018 | 0.030 |
| **BOD** | 0.006 | 0.434 | 0.071 | 0.636 | 0.056 |  | 0.033 | 0.089 | 0.012 | 0.035 | 0.012 | 0.017 | 0.021 |
| **LUZ** | 0.004 | 0.152 | 0.031 | 0.633 | 0.053 | 0.022 |  | 0.021 | 0.011 | 0.046 | 0.041 | 0.024 | 0.009 |
| **MIM** | 0.004 | 0.068 | 0.048 | 0.514 | 0.008 | 0.004 | 0.012 |  | 0.028 | 0.348 | 0.052 | 0.009 | 0.046 |
| **CAP** | 0.003 | 0.235 | 0.008 | 0.598 | 0.026 | 0.007 | 0.014 | 0.024 |  | 0.349 | 0.107 | 0.086 | 0.046 |
| **PNI** | 0.001 | 0.117 | 0.016 | 0.063 | 0.015 | 0.005 | 0.029 | 0.134 | 0.070 |  | 0.107 | 0.016 | 0.043 |
| **SAB** | 0.002 | 0.099 | 0.062 | 0.187 | 0.009 | 0.007 | 0.007 | 0.022 | 0.064 | 0.316 |  | 0.061 | 0.044 |
| **SCA** | 0.002 | 0.099 | 0.009 | 0.299 | 0.006 | 0.006 | 0.007 | 0.035 | 0.068 | 0.344 | 0.107 |  | 0.043 |
| **SUM** | 0.002 | 0.198 | 0.010 | 0.355 | 0.031 | 0.010 | 0.010 | 0.186 | 0.076 | 0.344 | 0.105 | 0.072 |  |

**Table S8** Credibility interval (95%) of the number of migrants per generation (see Table S7 for the number of migrants) for the 17 populations of *Tabebuia serratifolia* in Brazil, based on Bayesian coalescent analysis. Credibility interval for migration is from populations in the rows into populations in the columns.

|  | **CCM** | | **CRA** | | **POF** | | **ALT** | | **ARA** | | **BOD** | | **LUZ** | | **MIM** | | **CAP** | | **PNI** | | **SAB** | |  | **SCA** |  | **SUM** | |
| --- | --- | --- | --- | --- | --- | --- | --- | --- | --- | --- | --- | --- | --- | --- | --- | --- | --- | --- | --- | --- | --- | --- | --- | --- | --- | --- | --- |
| **CCM** |  |  | 0.001 | 0.718 | 0.001 | 0.089 | 0.008 | 0.712 | 0.000 | 0.064 | 0.001 | 0.027 | 0.000 | 0.059 | -0.001 | 0.242 | 0.000 | 0.084 | -0.002 | 0.376 | 0.000 | 0.117 | 0.000 | | 0.051 | 0.002 | 0.000 |
| **CRA** | 0.000 | 0.006 |  |  | 0.000 | 0.088 | -0.004 | 0.700 | 0.000 | 0.063 | 0.000 | 0.027 | 0.000 | 0.058 | -0.002 | 0.241 | 0.001 | 0.083 | -0.005 | 0.362 | -0.001 | 0.117 | 0.000 | | 0.051 | 0.000 | 0.051 |
| **POF** | 0.001 | 0.006 | 0.001 | 0.711 |  |  | 0.005 | 0.706 | 0.000 | 0.064 | 0.000 | 0.027 | 0.000 | 0.058 | -0.002 | 0.241 | 0.000 | 0.083 | -0.003 | 0.375 | 0.000 | 0.117 | 0.000 | | 0.051 | 0.000 | 0.051 |
| **ALT** | 0.000 | 0.000 | -0.007 | 0.000 | 0.000 | 0.000 |  |  | 0.000 | 0.000 | 0.000 | 0.000 | 0.000 | 0.000 | 0.004 | 0.000 | 0.001 | 0.000 | -0.003 | 0.000 | 0.000 | 0.000 | 0.002 | | 0.000 | 0.000 | 0.051 |
| **ARA** | 0.001 | 0.006 | -0.001 | 0.712 | 0.000 | 0.089 | 0.001 | 0.709 |  |  | 0.001 | 0.015 | 0.002 | 0.059 | 0.002 | 0.241 | 0.000 | 0.083 | -0.004 | 0.375 | -0.001 | 0.118 | 0.000 | | 0.051 | 0.000 | 0.051 |
| **BOD** | 0.001 | 0.006 | -0.002 | 0.715 | 0.000 | 0.089 | 0.064 | 0.713 | 0.000 | 0.064 |  |  | 0.000 | 0.058 | 0.001 | 0.243 | 0.000 | 0.083 | -0.002 | 0.376 | 0.000 | 0.117 | 0.000 | | 0.051 | 0.000 | 0.051 |
| **LUZ** | 0.000 | 0.006 | -0.003 | 0.709 | 0.000 | 0.087 | 0.020 | 0.711 | 0.000 | 0.064 | 0.000 | 0.027 |  |  | -0.002 | 0.240 | 0.000 | 0.084 | -0.004 | 0.373 | 0.000 | 0.117 | 0.000 | | 0.051 | 0.003 | 0.052 |
| **MIM** | 0.000 | 0.006 | -0.004 | 0.710 | 0.001 | 0.089 | 0.002 | 0.706 | 0.000 | 0.063 | 0.000 | 0.027 | 0.000 | 0.058 |  |  | 0.000 | 0.083 | 0.010 | 0.382 | 0.000 | 0.117 | 0.003 | | 0.052 | 0.000 | 0.051 |
| **CAP** | 0.000 | 0.006 | -0.003 | 0.711 | 0.001 | 0.088 | 0.003 | 0.706 | 0.000 | 0.063 | 0.000 | 0.027 | 0.000 | 0.058 | -0.002 | 0.242 |  |  | 0.014 | 0.383 | 0.005 | 0.119 | 0.001 | | 0.052 | 0.000 | 0.051 |
| **PNI** | 0.000 | 0.006 | -0.006 | 0.705 | 0.000 | 0.088 | 0.006 | 0.698 | 0.000 | 0.063 | 0.000 | 0.027 | 0.000 | 0.058 | 0.001 | 0.243 | 0.003 | 0.084 |  |  | 0.006 | 0.119 | 0.000 | | 0.051 | 0.003 | 0.052 |
| **SAB** | 0.000 | 0.006 | -0.003 | 0.707 | -0.001 | 0.089 | -0.004 | 0.702 | 0.000 | 0.063 | 0.000 | 0.027 | 0.000 | 0.058 | -0.003 | 0.241 | 0.001 | 0.084 | 0.003 | 0.382 |  |  | 0.003 | | 0.052 | 0.000 | 0.051 |
| **SCA** | 0.000 | 0.006 | -0.006 | 0.708 | 0.000 | 0.088 | -0.001 | 0.704 | 0.000 | 0.063 | 0.000 | 0.027 | 0.000 | 0.058 | -0.001 | 0.241 | 0.000 | 0.084 | 0.004 | 0.381 | 0.004 | 0.118 |  | |  | 0.000 | 0.051 |
| **SUM** | 0.000 | 0.006 | -0.004 | 0.710 | 0.000 | 0.088 | -0.001 | 0.707 | 0.000 | 0.063 | 0.000 | 0.027 | 0.000 | 0.058 | 0.000 | 0.244 | 0.000 | 0.084 | 0.007 | 0.383 | 0.001 | 0.118 | 0.000 | | 0.051 |  |  |

**Table S9** Uncertainty of the modelling components from ecological niche modelling predictions for *Tabebuia serratifolia* as revealed by hierarchical ANOVA. SS: sum of square.

| **Source of variation** | **Median SS** | **Minimun** | **Maximum** |
| --- | --- | --- | --- |
| **TIME** | 0.054 | 0.000 | 0.666 |
| **AOGCM** | 0.180 | 0.007 | 0.708 |
| **ENM** | 0.329 | 0.039 | 0.859 |
| **AOGCM x ENM** | 0.051 | 0.004 | 0.162 |

**Tabela S10** Classification of 60 predictive maps according to five biogeographical hypotheses across time: (1) PLAH; (2) PPPH; (3) Both (PLAH+PPPH); (4) None and (5) Retraction.

| **AOGCM** | **ENM** | **Range Size** | | **Range Shift** | |
| --- | --- | --- | --- | --- | --- |
|  |  | **0 ka** | **21 ka** | **0 - 21 ka** | **H** |
| CCSM | BioClim | 3220 | 2793 | 427 | 3 |
| CCSM | ENFA | 3418 | 3244 | 174 | 2 |
| CCSM | EuclidDist | 3021 | 2862 | 159 | 3 |
| CCSM | FDA | 2695 | 2594 | 101 | 2 |
| CCSM | GAM | 2705 | 2957 | -252 | 2 |
| CCSM | GLM | 2684 | 2981 | -297 | 2 |
| CCSM | GowerDist | 3474 | 3219 | 255 | 3 |
| CCSM | MahalanobisDist | 3189 | 2807 | 382 | 2 |
| CCSM | MARS | 2770 | 2816 | -46 | 2 |
| CCSM | MaxEnt | 2541 | 2665 | -124 | 2 |
| CCSM | NNet | 3720 | 3073 | 647 | 2 |
| CCSM | RndFor | 2923 | 2949 | -26 | 4 |
| CNRM | BioClim | 2751 | 2776 | -25 | 2 |
| CNRM | ENFA | 3022 | 2756 | 266 | 2 |
| CNRM | EuclidDist | 2924 | 2881 | 43 | 4 |
| CNRM | FDA | 2731 | 3058 | -327 | 2 |
| CNRM | GAM | 2588 | 3082 | -494 | 3 |
| CNRM | GLM | 2736 | 3509 | -773 | 3 |
| CNRM | GowerDist | 3039 | 2961 | 78 | 4 |
| CNRM | MahalanobisDist | 3260 | 3174 | 86 | 4 |
| CNRM | MARS | 2873 | 3184 | -311 | 2 |
| CNRM | MaxEnt | 2165 | 2607 | -442 | 2 |
| CNRM | NNet | 3258 | 2857 | 401 | 2 |
| CNRM | RndFor | 2577 | 2853 | -276 | 4 |
| MIROC | BioClim | 2986 | 2989 | -3 | 2 |
| MIROC | ENFA | 3055 | 3229 | -174 | 2 |
| MIROC | EuclidDist | 2995 | 2979 | 16 | 3 |
| MIROC | FDA | 2858 | 2737 | 121 | 2 |
| MIROC | GAM | 2663 | 2759 | -96 | 2 |
| MIROC | GLM | 2716 | 3055 | -339 | 2 |
| MIROC | GowerDist | 3088 | 3096 | -8 | 3 |
| MIROC | MahalanobisDist | 3063 | 3080 | -17 | 2 |
| MIROC | MARS | 2907 | 2968 | -61 | 2 |
| MIROC | MaxEnt | 2421 | 2597 | -176 | 2 |
| MIROC | NNet | 3018 | 2332 | 686 | 2 |
| MIROC | RndFor | 3078 | 3099 | -21 | 2 |
| MPI | BioClim | 2530 | 2457 | 73 | 2 |
| MPI | ENFA | 3242 | 2989 | 253 | 2 |
| MPI | EuclidDist | 2737 | 2712 | 25 | 3 |
| MPI | FDA | 2592 | 2536 | 56 | 5 |
| MPI | GAM | 2304 | 2959 | -655 | 2 |
| MPI | GLM | 2385 | 2998 | -613 | 2 |
| MPI | GowerDist | 2615 | 2608 | 7 | 3 |
| MPI | MahalanobisDist | 2726 | 2656 | 70 | 2 |
| MPI | MARS | 2537 | 3106 | -569 | 2 |
| MPI | MaxEnt | 2361 | 2615 | -254 | 2 |
| MPI | NNet | 2694 | 2763 | -69 | 2 |
| MPI | RndFor | 2628 | 2933 | -305 | 2 |
| MRI | BioClim | 3200 | 2994 | 206 | 2 |
| MRI | ENFA | 3282 | 2636 | 646 | 2 |
| MRI | EuclidDist | 3058 | 2755 | 303 | 2 |
| MRI | FDA | 2959 | 3013 | -54 | 5 |
| MRI | GAM | 3006 | 3448 | -442 | 2 |
| MRI | GLM | 2878 | 2460 | 418 | 5 |
| MRI | GowerDist | 3108 | 2492 | 616 | 2 |
| MRI | MahalanobisDist | 3212 | 2675 | 537 | 2 |
| MRI | MARS | 3081 | 2735 | 346 | 5 |
| MRI | MaxEnt | 2715 | 2641 | 74 | 2 |
| MRI | NNet | 3366 | 1707 | 1659 | 5 |
| MRI | RndFor | 2706 | 2766 | -60 | 5 |

*"H"* – biogeographical hypothesis for map classification.

**Table S11** Profile of haplotype (*h*) diversity and nucleotide (π) diversity obtained from the 2,000 simulations of five different demographical scenarios using the software BayeSSC, for chloroplast and ITS sequences. Observed genetic parameter means for chloroplast DNA, *h* = 0.584, π = 0.0018; for ITS, *h* = 0.337, π = 0.0022.

|  | **Chloroplast** | | | | **ITS** | | | |
| --- | --- | --- | --- | --- | --- | --- | --- | --- |
| **Models** | *h* | | π | | *h* | | π | |
|  | ***Range*** | ***Mean (SD)*** | ***Range*** | ***Mean (SD)*** | ***Range*** | ***Mean (SD)*** | ***Range*** | ***Mean (SD)*** |
| **PLAH** | 0.010 – 0.630 | 0.048 (0.115) | 6.4E-6 – 0.0026 | 2.9E-5 (6.4E-5) | 0.010 – 0.592 | 0.409 (0.103) | 2.3E-5 – 0.0028 | 8.1E-5 (0.0002) |
| **PPPH** | 0.010 – 0.743 | 0.090 (0.160) | 6.4E-6 – 0.0028 | 5.8E-5 (0.0001) | 0.010 – 0.691 | 0.083 (0.156) | 2.3E-5 – 0.0029 | 0.0018 (0.0004) |
| **Both** | 0.011 – 0.662 | 0.040 (0.101) | 6.4E-6 – 0.0029 | 2.4E-5 (6.5E-5) | 0.010 – 0.574 | 0.042 (0.106) | 2.0E-5 – 0.0030 | 8.5E-5 (0.0002) |
| **Retraction** | 0.002 – 0.658 | 0.067 (0.143) | 1.5E-6 – 0.0020 | 4.1E-5 (9.1E-5) | 0.002 – 0.698 | 0.063 (0.139) | 3.5E-6 – 0.0029 | 0.0001 (0.0002) |
